# Weight-bearing activity impairs nuclear membrane and genome integrity via YAP activation in plantar melanoma

**DOI:** 10.1038/s41467-022-29925-x

**Published:** 2022-04-25

**Authors:** Jimyung Seo, HyunSeok Kim, Kyoung Il Min, Changgon Kim, Yongsoo Kwon, Zhenlong Zheng, Yusung Kim, Hyung-Soon Park, Young Seok Ju, Mi Ryung Roh, Kee Yang Chung, Joon Kim

**Affiliations:** 1 Graduate School of Medical Science and Engineering, Korea Advanced Institute of Science and Technology (KAIST), Daejeon, Korea; 2 Department of Dermatology, Severance Hospital, Cutaneous Biology Research Institute, Yonsei University College of Medicine, Seoul, Korea; 3 Department of Mechanical Engineering, Korea Advanced Institute of Science and Technology (KAIST), Daejeon, Korea; 4 Department of Dermatology, Gangnam Severance Hospital, Cutaneous Biology Research Institute, Yonsei University College of Medicine, Seoul, Korea

**Keywords:** Melanoma, Melanoma, Cell signalling, Genomic instability

## Abstract

Acral melanoma commonly occurs in areas that are not exposed to much sunlight, such as the sole of the foot. Little is known about risk factors and mutational processes of plantar acral melanoma. Nuclear envelope rupture during interphase contributes to genome instability in cancer. Here, we show that the nuclear and micronuclear membranes of melanoma cells are frequently ruptured by macroscopic mechanical stress on the plantar surface due to weight-bearing activities. The marginal region of plantar melanoma nodules exhibits increased nuclear morphological abnormalities and collagen accumulations, and is more susceptible to mechanical stress than the tumor center. An increase in DNA damage coincides with nuclear membrane rupture in the tumor margin. Nuclear envelope integrity is compromised by the mechanosensitive transcriptional cofactor YAP activated in the tumor margin. Our results suggest a mutagenesis mechanism in melanoma and explain why plantar acral melanoma is frequent at higher mechanical stress points.

## Abstract (web summary)

Acral melanoma affects the soles of the feet but the etiology is unknown. Here, the authors show that melanoma cells implanted into the foot pad of mice and exposed to mechanical stress show features of nuclear rupture, DNA damage, and Yap activation.

## Introduction

Melanoma of the skin is characterized by a high mutational load due to solar UV radiation. However, in acral melanoma, which occurs mainly in areas with low UV exposure such as the sole of the foot, the mutational load is relatively low, but DNA structural variations are frequent^1,2^. Little is known about the mutational processes of acral melanoma. Acral melanoma is rare in Caucasian patients, but is the most common subtype of melanoma in Asian countries^3,4^. Anti-PD1 checkpoint inhibitor therapy has limited efficacy in patients with acral melanoma^5^. Elucidation of risk factors and mutation mechanisms is the key to advances in the prevention and treatment of acral melanoma. Acral melanoma frequently occurs at points of higher mechanical stress on the sole^6,7^, suggesting that mechanical stress contributes to the development of acral melanoma. Therefore, it is conceivable that weight-bearing activity is involved in the pathogenesis of plantar acral melanoma.

The nuclear envelope separates the nuclear genome from cytoplasmic components. Mechanical stress buffering through the nuclear lamina and lamina-associated chromatin is critical for maintaining nuclear envelope integrity^8,9^. Nuclear envelope rupture, which occurs more frequently in cancer cells than normal cells, is an important mechanism that promotes genome instability^10–12^. Recent studies have shown that mechanical stress applied to the nuclear envelope during cancer cell migration causes nuclear envelope rupture and DNA damage^13,14^. DNA structural variations, such as chromothripsis, are commonly caused by the rupture of micronuclei, extra-nuclear bodies generated by defects in mitosis, or nuclear reformation^15–17^. Although abnormal changes in size and shape of the nuclear envelope are well-established diagnostic features for identifying cancer cells, a potential link between morphological abnormalities and loss of nuclear membrane integrity has not been validated. It is also not known whether macroscopic mechanical stress resulting from weight load can be transmitted to cancer cells and disrupt the nuclear and micronuclear membranes.

Nuclear envelope rupture causes chromatin herniation, leading to the accumulation of cyclic GMP-AMP synthase (cGAS), a cytosolic DNA sensor, at the rupture site^14^. The interaction between cGAS and DNA activates the cGAS-STING pathway, a key component of the innate immune system^18^. Micronuclei, which commonly lack intact nucleoskeletal structures, are particularly prone to irreversible rupture, and thus cGAS localization to micronuclei is frequently observed in cancer cells^19^. The cGAS-STING pathway, which induces the expression of proinflammatory genes, has been shown to drive inflammation-mediated tumor progression and metastasis^18,20^. Nuclear envelope rupture exists only transiently and usually does not induce cell death, as cells rapidly repair nuclear membranes using components of the endosomal sorting complex required for transport III (ESCRT III) machinery^14^. However, nuclear envelope rupture causes persistent DNA damage dispersed throughout the nucleus, suggesting that diffusing cytoplasmic factors enter and induce DNA damage^15,21^.

Although not as unstable as micronuclei, the nuclear envelope of cancer cells also appears to be inherently susceptible to mechanical stress. Spontaneous nuclear envelope rupture occurs in several human cancer cell lines without overt lamin deficiency and external mechanical stress^22^. In addition, the loss of the two prototypical tumor suppressors, TP53 or RB, increases the frequency of nuclear envelope rupture^23^. Oncogenes that act as major contributors to nuclear envelope instability have not yet been identified. YAP is a transcriptional cofactor that is activated in several human solid tumors to promote cancer initiation, sustained proliferation, and metastasis^24,25^. In melanoma, YAP is known to induce resistance to BRAF inhibitor therapy^26,27^. Various regulatory factors that determine YAP activity have been identified, among which the Hippo pathway plays a central role as a YAP repressor^28,29^. Higher extracellular matrix stiffness and mechanical forces applied to the cell strongly activate YAP through their effect on the architecture of the contractile actin cytoskeleton^30,31^. Transcriptional targets of YAP include actin polymerization regulators and extracellular matrix components, suggesting that YAP mediates feedback regulation of cellular mechanotransduction^27,32^. Contractile actin filaments that increase nuclear pressure contribute to nuclear envelope rupture^33^. Thus, YAP-dependent mechanotransduction is a candidate for mediating the link between weight load and nuclear membrane instability in cancer.

Using an orthotopic implantation mouse model, we show that macroscopic mechanical stress from weight-bearing activity damages the nuclear membranes and promotes genome instability in plantar melanoma. Mechanistically, activation of YAP by mechanical stress weakens the integrity of the nuclear envelope. Then, sustained mechanical stress due to weight-bearing activity disrupts the weakened nuclear and micronuclear membranes, which activates cytosolic cGAS. YAP activation and nuclear envelop rupture were observed concurrently with nuclear morphological abnormalities in the marginal region of the plantar melanoma both in mice and human. Our data suggest that weight-bearing activity can impair nuclear membrane and genome integrity via YAP activation in plantar melanoma.

## Results

### DNA damage and nuclear membrane rupture are frequent in the marginal region of plantar melanoma nodules

**Fig. 1:**
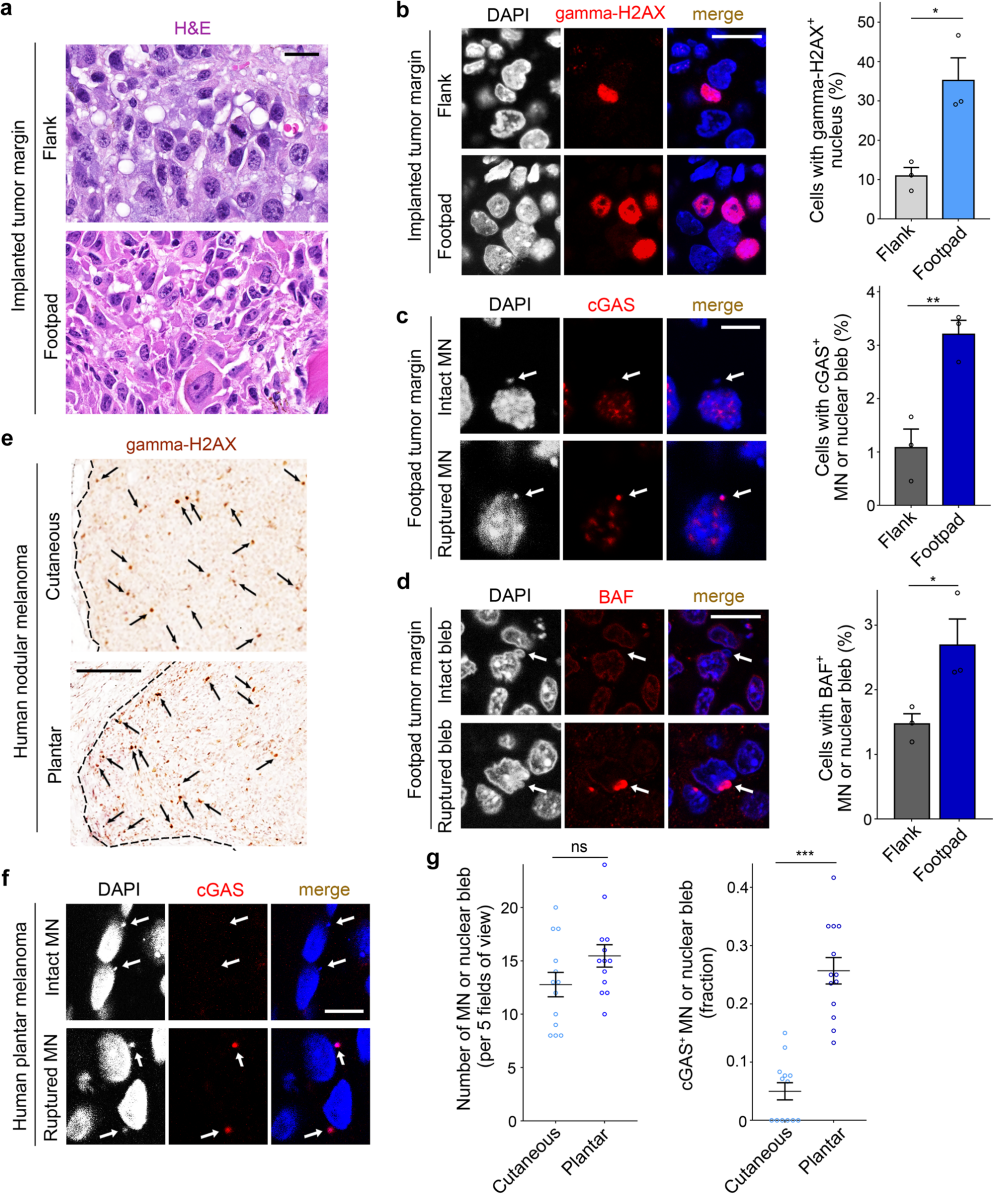
DNA damage and nuclear membrane rupture in the marginal region of plantar melanoma. **a** Nuclear morphology in the tumor margin. B16F10 cells were simultaneously implanted into the flank skin and the footpad of C56BL/6J mice. The images shown are representative results of three mice. **b** Labeling of tumor sections with gamma-H2AX antibody, and quantification of nuclear gamma-H2AX^+^ cells (*n* = 3 mice; *P = 0.0165). **c** Detection and quantification of micronucleus (MN)/nuclear bleb rupture using DAPI and anti-cGAS antibody (*n* = 3 mice; **P = 0.0082). **d** Detection and quantification of MN/nuclear bleb rupture using DAPI and anti-BAF antibody (*n* = 3 mice; *P = 0.0481). **e** Detection of gamma-H2AX^+^ cells (arrows) in human nodular melanomas resected from the cheek (cutaneous) and the heel of the foot (plantar). The images shown are representative results of three melanoma samples per group. **f** Intact or ruptured MN in human plantar melanoma. The images shown are representative results of thirteen plantar melanoma samples. **g** The number of MN/nuclear blebs and the fraction of ruptured MN/nuclear blebs (*n* = 13 human melanomas per group; ***P < 0.0001). Each dot on the graphs represents data obtained from one mouse (**b**, **c** and **d**) and one patient (**g**). Error bars are SEM (two-tailed unpaired *t* test). ns, not significant. Size bars: 20 μm (**a**, **b**), 10 μm (**c**, **d** and **f**), and 200 μm (**e**). Source data are provided as a Source Data file.

The degree of weight loading varies depending on the body part, and the sole of the foot is the part that receives the strongest load on the body surface. To analyze the effect of weight load on melanoma progression while ruling out genetic differences in melanoma subtypes, we simultaneously implanted B16F10 melanoma cells into the flank skin and hind footpad of C57BL/6J mice. Tumors formed by the implanted melanoma cells were examined after 13 days. A high level of nuclear morphological irregularity was observed in the marginal region of the footpad tumors, whereas relatively few nuclear morphological abnormalities were observed in the center of footpad tumors as well as in the flank skin tumors (Fig. 1a and Supplementary Fig. 1a). This subjective observation was confirmed by measurements of the compactness and circularity of the nucleus (Supplementary Fig. 1b). In this study, the tumor margin was defined as a tumor area 250 μm wide at the interface with the normal tissue. Cancer cells in the marginal region of footpad tumors showed higher levels of DNA damage detected by gamma-H2AX antibody staining compared to skin tumor margins^34^ (Fig. 1b). To determine whether the nuclear membrane is damaged, antibodies to cGAS and barrier-to-autointegration factor (BAF), which are known to be recruited to the rupture site of the nuclear membrane^14,35^, were used as markers. We observed an increase in the rupture of nuclear and micronuclear membranes in the footpad tumor margin as indicated by cGAS accumulation (Fig. 1c). Immunofluorescence for BAF also showed increased nuclear membrane rupture in the footpad tumor margin (Fig. 1d). These results suggest that the shape and integrity of the nuclear membrane in melanoma cells are influenced by the tumorigenic site and the intratumoral location.

In contrast to the flank skin, tumor growth was limited in the footpad, which may be due to spatial constraints. (Supplementary Fig. 1c). To test the influence of spatial constraints on nuclear morphology and DNA damage, we implanted B16F10 cells into the mouse tongue. The rate of tumor growth in the tongue was similar to that of the footpad (Supplementary Fig. 1c). However, cancer cells at the margin of the tongue tumors showed no significant differences in nuclear morphology and DNA damage compared to the center (Supplementary Fig. 1b, d). We next tested whether T cell-mediated immune responses are related to the phenotype detected in the footpad tumor. We observed an increase in nuclear shape irregularity in the margin of implanted footpad tumors from *Foxn1* mutant (nude) mice defective in T cell-mediated immunity^36^ (Supplementary Fig. 2a). Also, the number of nuclei and micronuclei stained with gamma-H2AX antibody increased significantly in the margin of the footpad tumors in *Foxn1* mutant mice (Supplementary Fig. 2b). These results rule out the possibility that spatial constraints or T cell-mediated immunity may underlie the phenotype of the footpad tumor margin.

Next, we examined tumor nodule sections from 26 melanoma patients (Supplementary Table 1). To determine whether DNA damage is influenced by the tumorigenic site and the location of cancer cells within the tumor, we compared the frequency and distribution of cells expressing gamma-H2AX in subcutaneous melanoma nodules. DNA damage in plantar melanoma was more frequent in the marginal region, whereas skin melanoma of the upper body showed a sporadic distribution of cells labeled with gamma-H2AX antibody irrespective of location (Fig. 1e and Supplementary Fig. 3). We also examined the integrity of the nuclear and micronuclear membranes in the margin of melanoma nodules. The fraction of micronuclei and nuclear blebs stained with anti-cGAS antibody was significantly higher in plantar melanoma than in skin melanoma (Fig. 1f, g). The total number of micronuclei and nuclear blebs did not differ significantly (Fig. 1g). These observations suggest that nuclear membrane instability and DNA damage more frequent in the tumor margin are characteristics of plantar melanoma.

### Weight-bearing activity induces DNA damage and nuclear membrane rupture in mouse footpad tumors

**Fig. 2:**
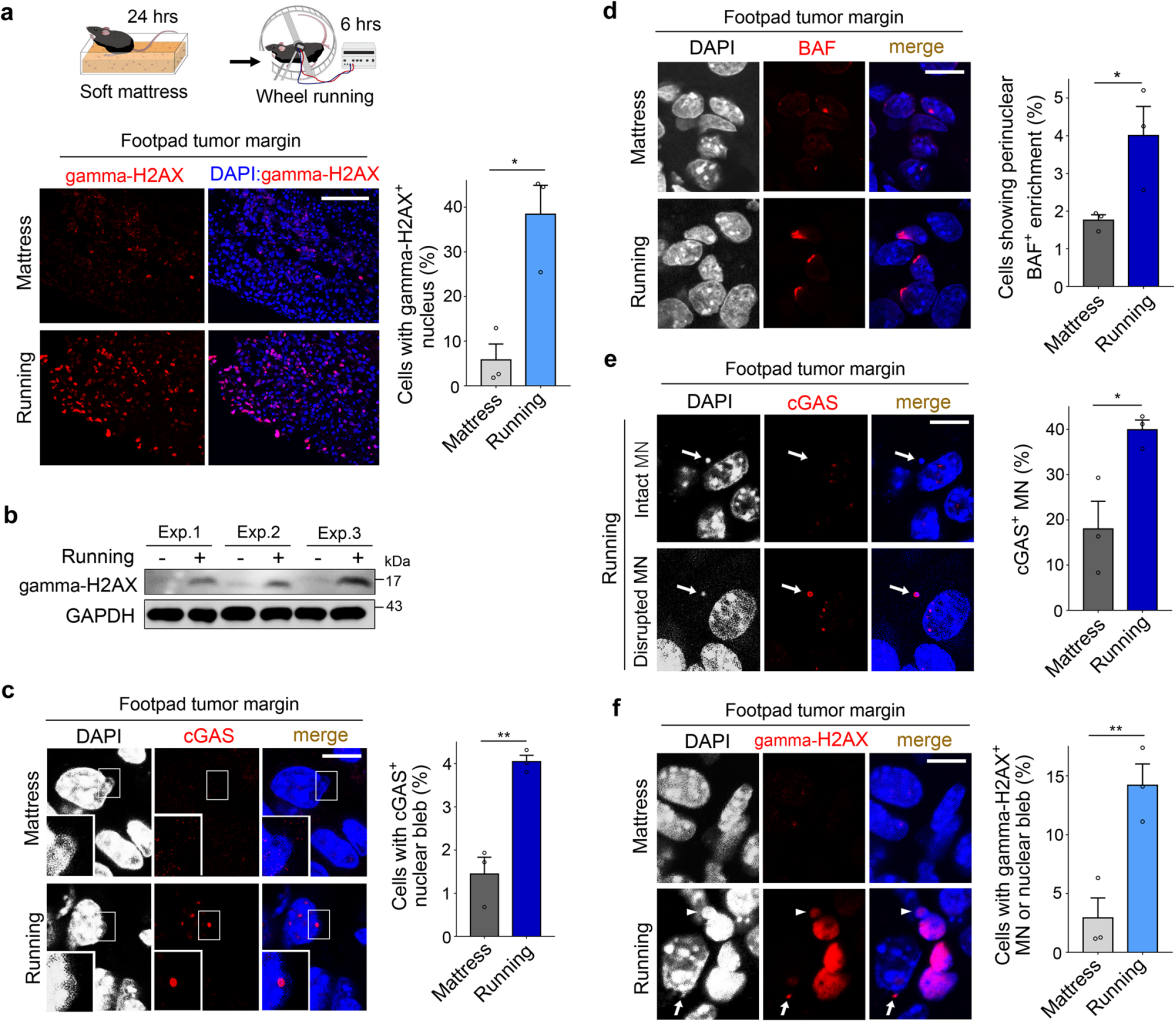
DNA damage and nuclear membrane rupture in mouse footpad tumors due to weight-bearing activity. **a** Detection and quantification of nuclear gamma-H2AX^+^ in the marginal region of footpad tumors after 6 h of forced wheel running (*n* = 3 mice per group; *P = 0.0116). Mice were placed on a soft latex mattress for 24 h before running. Image tiles are shown. **b** Immunoblot analysis of gamma-H2AX and GAPDH in footpad tumors after 6 h of forced wheel running (*n* = 3 mice per group). **c** Detection and quantification of cGAS^+^ nuclear blebs in the margin of footpad tumors after 6 h of forced wheel running (*n* = 3 mice per group; **P = 0.0033). **d** Detection and quantification of perinuclear BAF enrichment in the margin of footpad tumors after 6 h of forced wheel running (*n* = 3 mice per group; *P = 0.0462). **e** Detection and quantification of cGAS^+^ micronuclei (MN) in the margin of footpad tumors after 6 h of forced wheel running (*n* = 3 mice per group; *P = 0.0277). **f** Detection and quantification of gamma-H2AX^+^ MN/nuclear blebs in the margin of footpad tumors after 6 h of forced wheel running (*n* = 3 mice per group; *P = 0.0106). Each dot on the graphs represents data obtained from one mouse. Error bars are SEM (two-tailed unpaired t test). Size bars: 100 μm (**a**) and 10 μm (**c**–**f**). Source data are provided as a Source Data file.

To directly assess the impact of macroscopic mechanical stress on cancer cells due to weight bearing activity, we established a mouse model that applies mechanical stress to tumors implanted in the footpads. Mice with a footpad tumor were placed on a soft latex mattress (density: 50 kg/m^3^) for 24 h to minimize mechanical irritation of the footpad, and then forced wheel running was performed (Supplementary Fig. 4a). The number of cells exhibiting high levels of gamma-H2AX increased slightly after wheel running for 2 h (Supplementary Fig. 4b). Remarkably, a significant increase in gamma-H2AX positive cells was observed after a total of 6 h of running (Fig. 2a and Supplementary Fig. 4b). Cells exhibiting gamma-H2AX immunostaining were mainly localized in the tumor margin. Upregulation of gamma-H2AX due to mechanical stress was confirmed by an immunoblot analysis (Fig. 2b). cGAS and BAF immunofluorescence showed that wheel running promotes the rupture of nuclear blebs (Fig. 2c, d). In addition, wheel running caused an increase in the percentage of ruptured micronuclei (Fig. 2e). DNA damage in the ruptured micronuclei and nuclear blebs was revealed by gamma-H2AX immunostaining (Fig. 2f). In normal tissues around the footpad tumor, nuclear envelope rupture or DNA damage was not induced by forced wheel running (Supplementary Fig. 4c). Nuclear envelope rupture after wheel running was also visualized by transmission electron microscopy as well as Lamin B1 immunofluorescence staining (Supplementary Fig. 5). These results suggest that mechanical stress from weight-bearing activity damages the nuclear and micronuclear membranes, which are particularly weakened in cancer cells in the tumor margin.

### Weight load promotes YAP activation in the margin of plantar melanoma

**Fig. 3:**
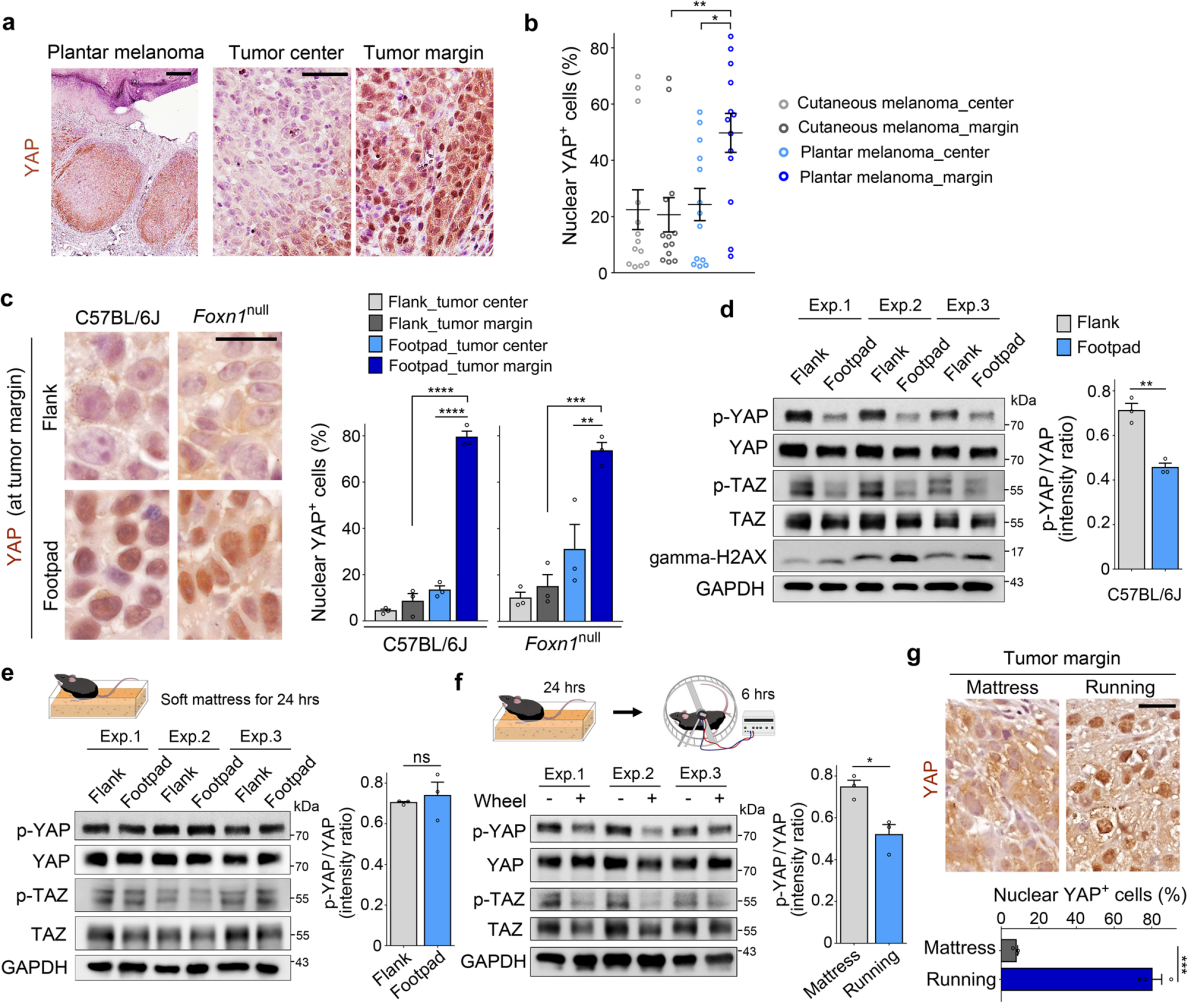
YAP activation in the marginal region of plantar melanoma. **a** Immunohistochemistry analysis of YAP in human plantar melanoma. **b** Quantification of cells exhibiting YAP nuclear enrichment in the center and margin of human cutaneous and plantar melanomas (*n* = 13 human melanomas per group; **P = 0.0135 and *P = 0.0377). **c** Detection and quantification of implanted B16F10 cells exhibiting YAP nuclear enrichment in C57BL/6J mice and *Foxn1*-null mice (*n* = 3 mice each; ****P < 0.0001, ***P = 0.0009 and **P = 0.0068). **d** Immunoblot analysis of the indicate proteins. The bar graph shows the intensity ratio of phosphorylated YAP (S127) and total YAP (*n* = 3 mice; **P = 0.0026). **e** Immunoblot analysis and quantification of band intensity (*n* = 3 mice). Mice with implanted tumors were placed on a soft latex mattress for 24 h before analysis. **f** Immunoblot analysis and quantification of band intensity (*n* = 3 mice per group; *P = 0.0175). Mice were placed on a soft latex mattress for 24 h and forced to run on the wheel for a total of 6 h. **g** Detection and quantification of cells exhibiting YAP nuclear enrichment in tumors prepared as in (**f**) (*n* = 3 mice per group; ***P = 0.0002). Each dot on the graphs represents data obtained from one patient (**b**) and one mouse (**c**–**g**). Error bars are SEM [one-way ANOVA with a Tukey’s post hoc test (**b**, **c**); two-tailed unpaired t test (**d**–**g**)]. Size bars: 200 μm (**a** left), 50 μm (**a** right), and 20 μm (**c**, **g**). Source data are provided as a Source Data file.

The mechanotransduction pathway in which YAP is activated by static mechanical stimuli that affect the actin cytoskeleton is well elucidated in cell culture models^37,38^. However, it has not been clearly proven whether macroscopic and dynamic mechanical stress from weight-bearing activities affects YAP in vivo. To address whether YAP activity is related to mechanical stress acting on plantar tumors, we performed YAP immunostaining on melanoma patient samples. Cells exhibiting high levels of nuclear YAP enrichment indicative of YAP activation were more frequent in the margin of plantar melanoma than in the tumor center (Fig. 3a and Supplementary Fig. 6a). In UV-induced skin melanoma, nuclear enrichment of YAP was not clearly observed at both the tumor margin and the tumor center (Fig. 3b and Supplementary Fig. 6a).

Similar to patient samples, nuclear enrichment of YAP was mainly observed in the marginal region of footpad tumors in both C57BL/6J and *Foxn1*-null mice, whereas YAP was detected primarily in the cytoplasm in the footpad tumor center and flank skin tumors (Fig. 3c). The level of S127 phosphorylation, which mediates YAP cytoplasmic retention^39^, was higher in flank skin tumors than in footpad tumors (Fig. 3d and Supplementary Fig. 6b). As reported to occur in human melanoma cells^27^, YAP knockdown caused a reduction of Myc protein levels in B16F10 cells (Supplementary Fig. 6c). Consistent with YAP activity, elevated levels of nuclear Myc immunohistochemical staining was observed in the footpad tumor margin (Supplementary Fig. 6d). When mice were placed on a soft latex mattress for 24 h, the level of YAP S127 phosphorylation in footpad tumors became similar to that of flank skin tumors (Fig. 3e). After forced wheel running for 6 h, YAP S127 phosphorylation decreased (Fig. 3f), and the number of cells exhibiting nuclear enrichment of YAP increased significantly (Fig. 3g). Taken together, these results indicate that YAP activity is dynamically regulated by macroscopic mechanical stress transmitted to cancer cells.

### Compressive load promotes nuclear membrane rupture and DNA damage in YAP-activated cells

**Fig. 4:**
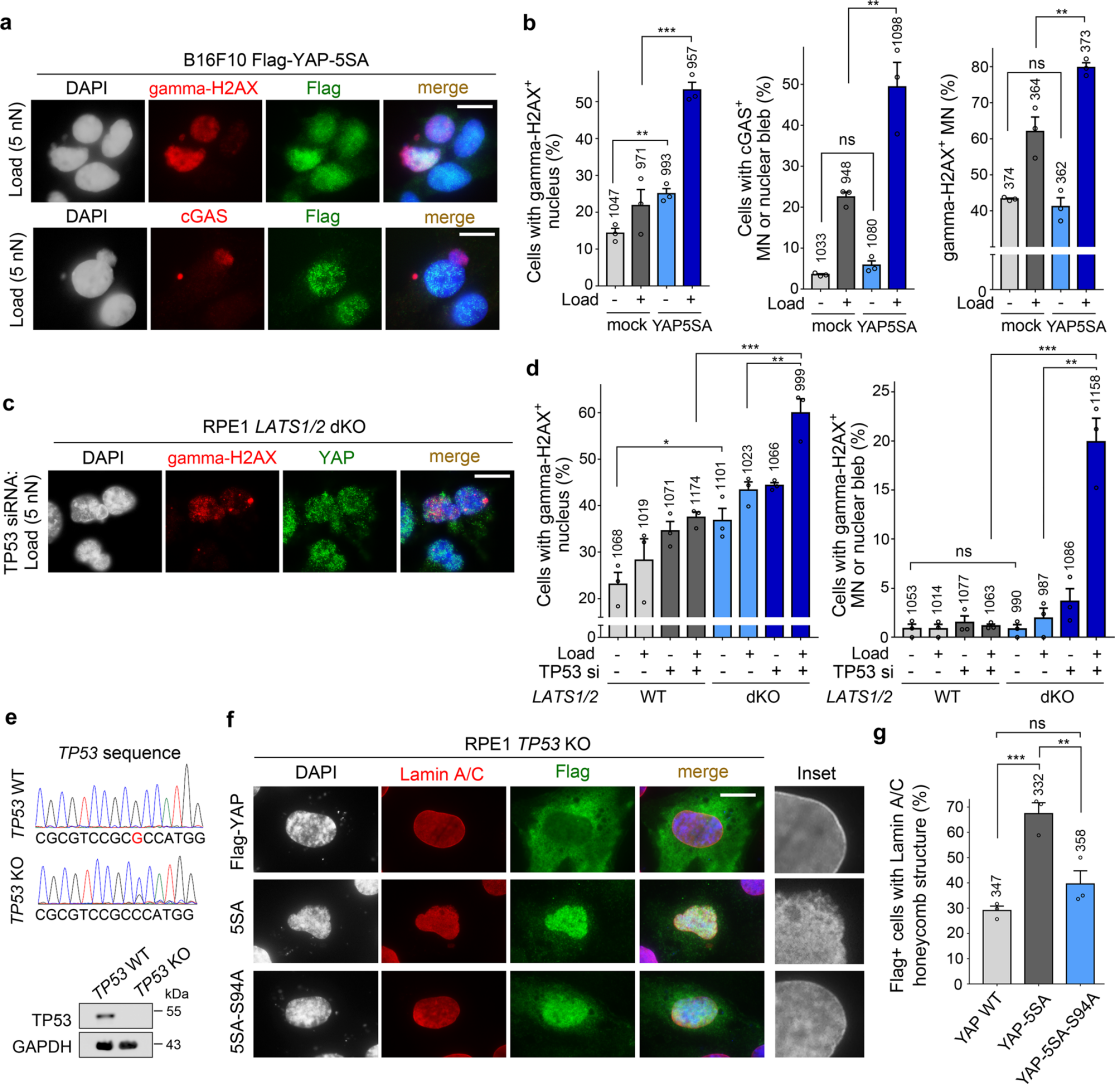
Compressive load-induced nuclear membrane rupture in YAP hyperactivated cells. **a** Gamma-H2AX and cGAS immunofluorescence in B16F10 cells stably expressing Flag-YAP-5SA. Cells were subjected to weight loading (5 nN) for 20 min. **b** Quantification of gamma-H2AX^+^ nuclei, cGAS^+^ micronuclei (MN)/nuclear blebs, and gamma-H2AX^+^ MN in mock transfected B16F10 cells and B16F10 Flag-YAP-5SA cells [*n* = 3 independent experiments; (left) **P = 0.0051 and ***P = 0.003; (middle) **P = 0.0120; (right) **P = 0.0140]. **c** Gamma-H2AX immunofluorescence in RPE1 LATS1/2 dKO cells subjected to weight loading for 20 min. Cells were transfected with TP53 siRNAs 3 days before weight loading. **d** Quantification of gamma-H2AX^+^ nuclei and gamma-H2AX^+^ MN/nuclear blebs [*n* = 3 independent experiments; (left) *P = 0.0201, **P = 0.0095 and ***P = 0.0024; (right) ***P = 0.0015 and **P = 0.0024]. **e** Confirmation of TP53 deletion. **f** Lamin A/C immunofluorescence in B16F10 cells 24 h after transfection of the indicated expression vectors. **g** Quantification of experimental results shown in (**f**) (*n* = 3 independent experiments; ***P = 0.0012 and **P = 0.0148). Error bars are SEM (two-tailed unpaired t test). Each dot represents data from one independent experiment. The number above each bar is the total number of cells or micronuclei (**b**, rightmost graph) analyzed. Size bars: 10 μm (**a**, **c** and **f**). Source data are provided as a Source Data file.

We next investigated whether YAP is a factor that makes the nuclear envelope of cancer cells susceptible to mechanical stress in the tumor margin. We established B16F10 and SKMEL28 cells stably expressing YAP5SA, a constitutive active form of YAP^39^, and performed in vitro weight loading experiments (Supplementary Fig. 7a, b). In the absence of a weight load, YAP5SA expression increased the number of B16F10 cells exhibiting nuclear gamma-H2AX staining, but did not change the number of cells exhibiting detectable spontaneous rupture of micronuclei or nuclear blebs (Fig. 4a, b). When weight was applied to B16F10-YAP5SA cells, not only DNA damage but also rupture of micronuclei and nuclear blebs were significantly increased compared to a mock transfected control (Fig. 4b and Supplementary Fig. 8a). In SKMEL28-YAP5SA cells, the number of cells with ruptured micronuclei or nuclear blebs increased even without a weight load (Supplementary Fig. 8b, c). YAP5SA expression in B16F10 and SKMEL28 cells did not affect the cell proliferation rate (Supplementary Fig. 8d, e). Additional human melanoma cells RPMI-7951 and A375SM expressing YAP5SA also showed increased susceptibility of the nuclear membrane to mechanical stress (Supplementary Fig. 8f–i). These results suggest that increased nuclear membrane instability is a common phenomenon induced by YAP hyperactivity in melanoma cell lines.

Loss of the tumor suppressors TP53 and RB has been reported to weaken the structural integrity of the nuclear envelope by an unknown mechanism^23^. We examined the effect of TP53 depletion in RPE1-LATS1/2dKO cells, where YAP is overactivated by inactivation of the *LATS1* and *LATS2* genes^40^ (Fig. 4c). In RPE1-LATS1/2 wildtype cells, TP53 knockdown and weight loading increased DNA damage, but there was no synergistic effect of the two treatments and the number of cells with detectable nuclear membrane rupture did not increase (Fig. 4d). In contrast, in RPE1-LATS1/2 dKO cells, TP53 knockdown and weight loading exerted a synergistic effect on both DNA damage and nuclear membrane rupture (Fig. 4d). Without TP53 knockdown, RPE1-LATS1/2 dKO cells showed elevated nuclear DNA damage compared to LATS1/2 wildtype cells, whereas spontaneous micronuclear or nuclear bleb rupture did not increase (Fig. 4d). To confirm the impact of TP53 loss and YAP activation on nuclear membrane instability, we edited *TP53* gene in RPE1 cells using CRISPR-Cas9 system. Homozygous 1-bp deletion and loss of TP53 expression were detected by sequencing and immunoblotting (Fig. 4e). Transfection with YAP-5SA vector caused an increase in the number of RPE1 *TP53* KO cells exhibiting a “honeycomb” pattern of Lamin A/C distribution indicative of nuclear lamina instability^41^, whereas YAP-5SA with S94A mutation, which abrogates its interaction with TEAD transcription factors^42^, did not promote nuclear lamina defects (Fig. 4f, g). Mechanical stress during transwell migration also promoted micronuclear and nuclear bleb rupture in B16F10 cells expressing YAP5SA (Supplementary Fig. 9a, b). In addition, EGFP-cGAS reporter expressed in RPE1-LATS1/2dKO cells showed that YAP activation and TP53 depletion synergistically promote nuclear membrane rupture during transwell migration (Supplementary Fig. 9c, d). Together, these results indicate that YAP activation contributes to the reduction of nuclear envelope integrity in cancerous cells.

To analyze the relationship between morphological abnormalities and susceptibility to mechanical stress of the nuclear envelope, we disrupted morphological homeostasis of the nucleus of RPE1-LATS/TP53 wildtype cells by depletion of the inner centromere protein INCENP (Supplementary Fig. 10a, b). Nuclear envelope rupture was detected by release of nuclear localization sequence-tagged EGFP (NLS-EGFP) into the cytoplasm. INCENP knockdown caused a prominent nuclear deformation, and the decrease in nuclear envelope integrity in INCENP-depleted cells was demonstrated by weight-loading experiments (Supplementary Fig. 10a, b). This observation suggests a potential association between morphology and membrane integrity of the nuclear envelope. In contrast to INCENP knockdown, YAP hyperactivity by LATS1/2 knockout did not induce apparent morphological changes in the nucleus (Supplementary Fig. 10c). Therefore, nuclear deformation does not appear to be the key mechanism by which YAP promotes nuclear membrane instability.

### Effect of nuclear envelop abnormalities on YAP nuclear localization

**Fig. 5:**
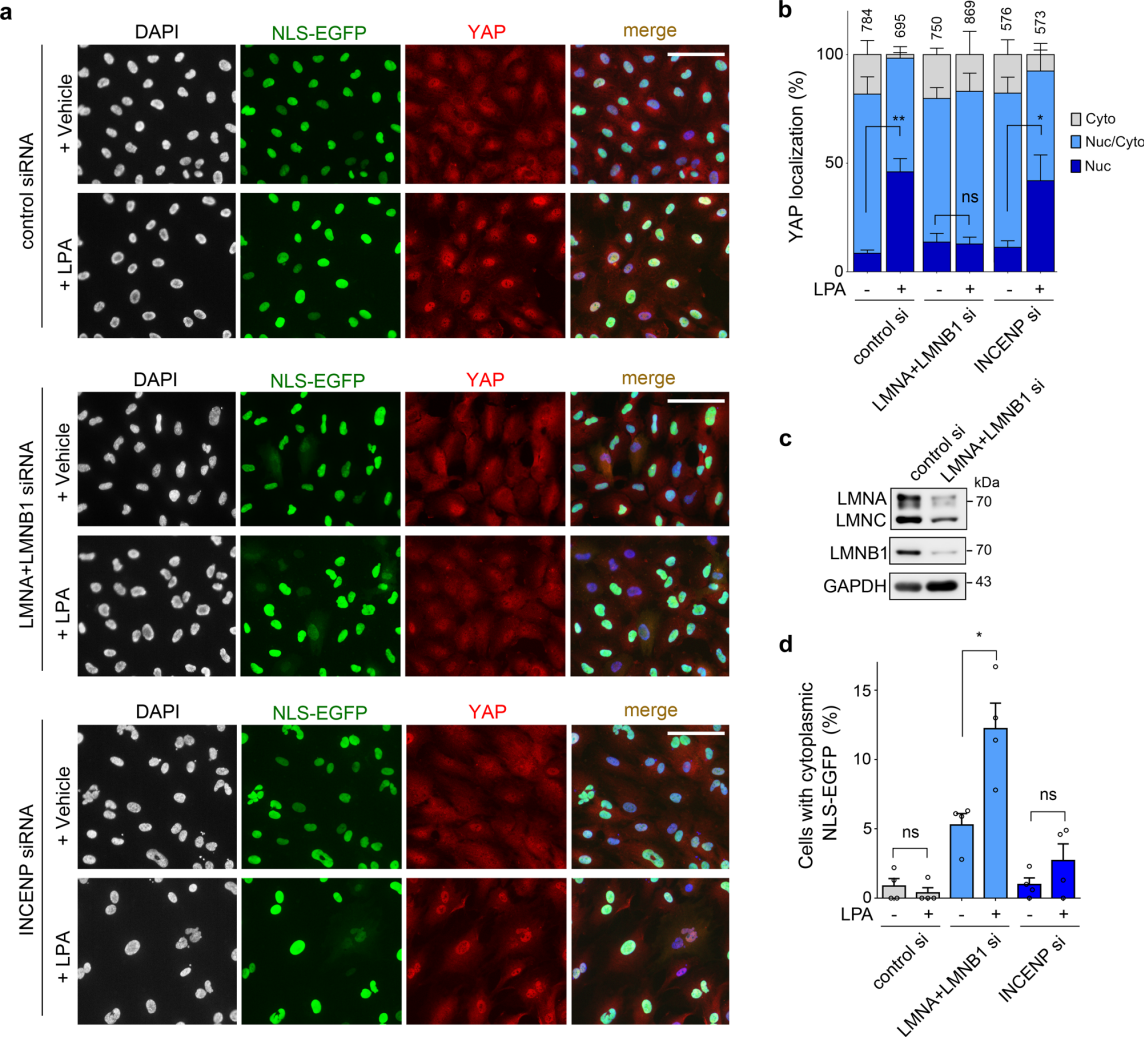
Effect of nuclear lamina defects and nuclear malformations on YAP nuclear translocation. **a** YAP immunofluorescence staining in RPE1 cells stably expressing NLS-EGFP after lamin (LMNA and LMNB1) or INCENP knockdown. Cells were treated with LPA (20 μM) for 30 min after 8 h serum starvation. **b** Quantification of the YAP localization pattern (Nuc, cells in which YAP is mainly observed inside the nucleus; Nuc/Cyto, equivalent YAP distribution in the nucleus and cytoplasm; and Cyto, cytoplasmic localization of YAP) (*n* = 4 independent experiments; **P = 0.001 and *P = 0.0465). **c** Immunoblot analysis confirming knockdown of the lamin genes. Data shown are representative results of two independent experiments. **d** Quantification of nuclear envelope rupture by monitoring the release of NLS-EGFP from the nucleus (*n* = 4 independent experiments; *P = 0.0139). Error bars are SEM (two-tailed unpaired t test). Each dot represents data from one independent experiment. The number above each bar is the total number of cells analyzed (**b** and **d** are quantifications for identical double-labeled cells). Size bars: 40 μm (**a**). Source data are provided as a Source Data file.

To determine whether nuclear envelope instability inversely affects YAP activity, we examined subcellular YAP distribution after disruption of nuclear envelope integrity by lamin depletion. In RPE1-NLS-EGFP cells transfected with non-targeting siRNAs, treatment of lysophosphatidic acid (LPA), which is known to activate YAP by inducing the formation of contractile actin filaments, promoted YAP nuclear localization (Fig. 5a, b). Similar to previous research results^43^, disruption of nuclear lamina by simultaneous knockdown of LMNA and LMNB1 genes inhibited LPA-induced YAP nuclear import (Fig. 5a–c). Contractile actin filament formation is known to contribute to nuclear envelope rupture by increasing nuclear pressure^33^. As expectedly, LPA treatment caused a significant increase in the number of cells exhibiting cytoplasmic leakage of NLS-EGFP in lamin-depleted cells (Fig. 5d). These results indicate that the lamina-defective nuclear envelope interferes with YAP nuclear import and is sensitive to actin-mediated mechanical stress. However, LPA-induced YAP nuclear localization was not affected by nuclear morphological abnormalities caused by INCENP depletion (Fig. 5a, b). Contrary to the response to weight loading (Supplementary Fig. 10a), the frequency of nuclear envelope rupture was not significantly increased by LPA treatment in cells depleted of INCENP. A quantitative difference in their impact on nuclear membrane integrity appears to exist between lamin and INCENP depletion, and weight loading seems to transmit a stronger mechanical stress to the nuclear envelope than LPA treatment.

Next, we examined the effect of weight loading on YAP localization. A weight load was applied to RPE1 cells at a high cell density condition, where YAP mainly localizes to the cytoplasm. Transient mechanical stress through weight loading did not induce nuclear translocation of YAP (Supplementary Fig. 11a). Under the same condition, LPA treatment promoted nuclear localization of YAP (Supplementary Fig. 11b). Staining of LPA-treated cells with dye-conjugated phalloidin revealed an increase in actin filament density throughout the cells (Supplementary Fig. 11b). Cells subjected to a weight load also exhibited an increase in actin filament density near the nucleus, but the level of increase was much lower than that observed in LPA-treated cells (Supplementary Fig. 11c). Although weight loading exerts a stronger mechanical stress on the nuclei of cell monolayers, it does not seem to induce actin cytoskeleton rearrangement sufficient to activate YAP. Remarkably, nuclear envelope rupture of B16F10 YAP-5SA cells due to weight loading was partially reduced by the actin destabilizer cytochalasin D (Supplementary Fig. 12a, b). This suggests that alterations in the dynamics of the actin cytoskeleton induced by weight loading indeed contribute to the rupture of the nuclear envelope.

### Deep sequencing revealed DNA damage newly acquired by RPE1 LATS1/2 dKO cells after implantation into nude mice

**Fig. 6:**
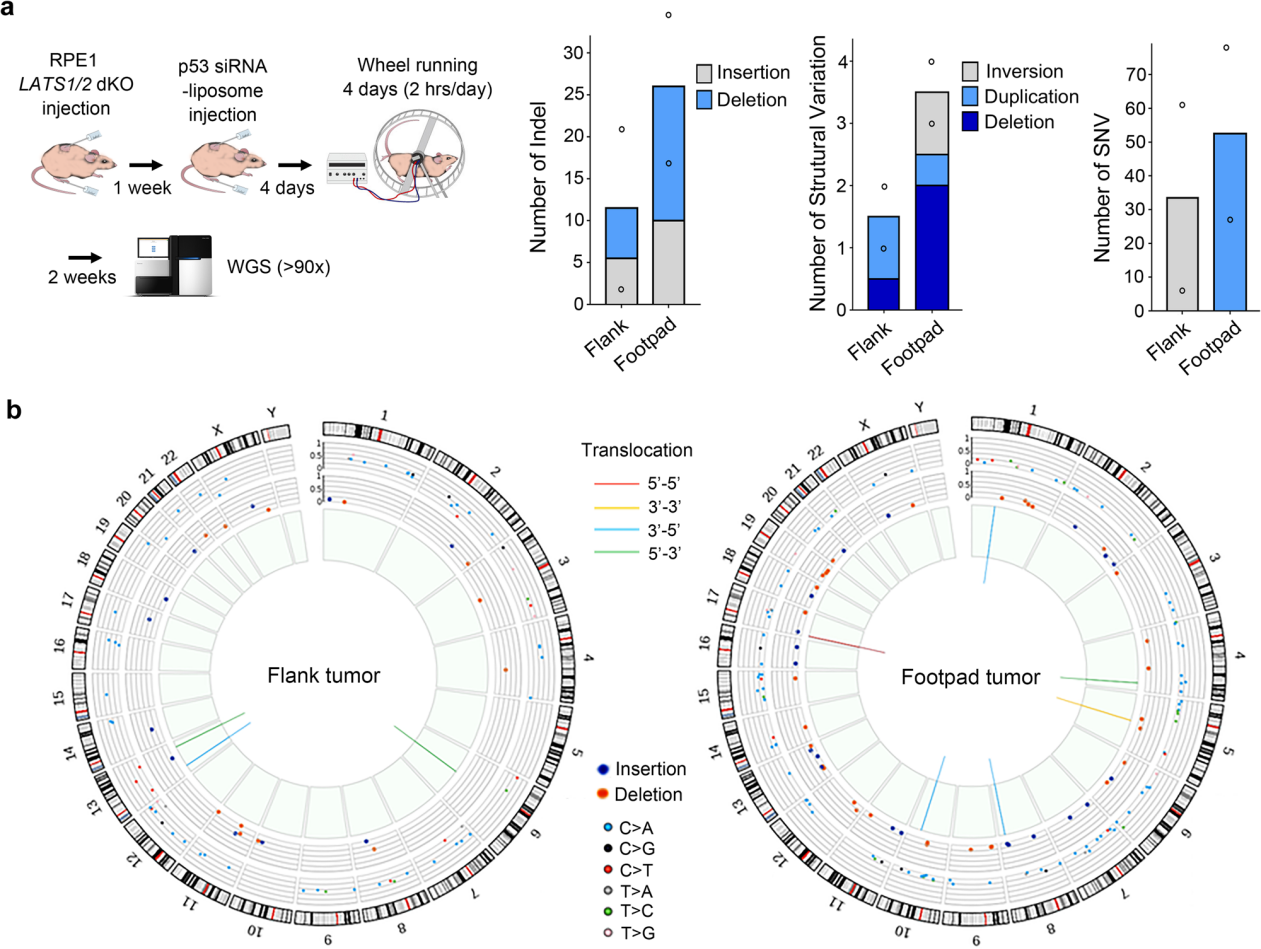
Whole genome sequencing analysis of RPE1 LATS1/2 dKO cells implanted into nude mice. **a** Deep whole-genome sequencing (WGS) analysis to detect genetic changes occurred in RPE1 LATS1/2 dKO cells implanted into the flank skin and the footpad (*n* = 2 mice). Cells were implanted into the flank skin and the footpad of nude mice simultaneously. Mice were subjected to wheel running for 4 days (2 h per day) after intratumoral injection of p53 siRNA-loaded liposomes. Tumors were analyzed 2 weeks after completion of wheel running. The genomic DNA sequence of RPE1 *LATS1/2*-dKO cells transfected with p53 siRNA was used as a reference. Indel: single nucleotide insertion and deletion. SNV: single nucleotide variation. **b** The circos plots show genome wide DNA alterations occurred in RPE1 LATS1/2 dKO cells after implantation into nude mice. Source data are provided as a Source Data file.

To demonstrate in vivo that DNA damage is induced in YAP-activated cells by weight-bearing activity, we performed whole genome DNA sequencing at high coverages (>90X). RPE1 LATS1/2-dKO cells were simultaneously implanted into the flank and footpad of Foxn1-null mice. To prevent cell death due to DNA damage, TP53 siRNA-loaded liposomes were injected into the developing tumor mass. Tumors were developed for 2 weeks after 4 days of wheel running (2 h per day). Genomic DNA extracted from fresh frozen tumor tissue was analyzed. The genomic DNA sequence of RPE1 LATS1/2-dKO cells transfected with TP53 siRNA was used as a reference. Intrachromosomal rearrangements, as well as single nucleotide insertions and deletions that were not originally present in RPE1 LATS1/2-dKO cells, were identified, and these alterations were more frequent in footpad tumors (Fig. 6). In addition, a higher number of single nucleotide variations were found in footpad tumors. These results suggest that tumors formed by YAP-activated RPE1 cells acquire more DNA variations, detectable even by bulk genome sequencing, under mechanical stress in a short period of time.

### YAP signature genes and inflammatory response genes are upregulated in mouse footpad tumors

**Fig. 7:**
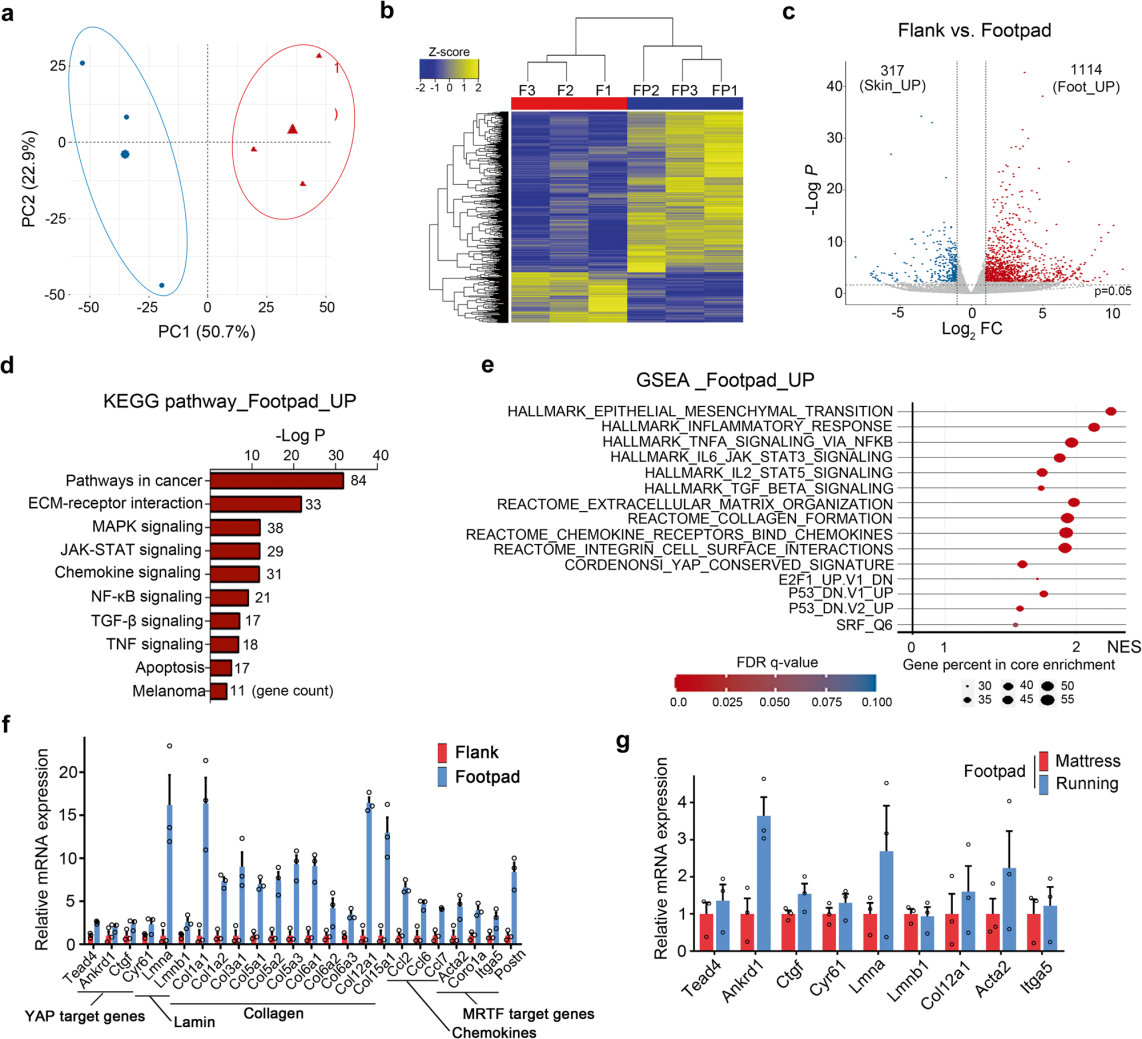
Transcriptome profiling of tumors generated in the flank and footpad. **a** Principal component analysis of RNAseq data for flank and footpad tumors. Each dot represents an individual tumor. **b** Heat map generated from clustering based on most differentially expressed genes [false discovery rate (FDR) < 0.05; F, flank tumors; FP, footpad tumors]. **c** Volcano plot showing the genes that are differentially expressed (*P* value < 0.05; fold change ≥ 2; red, higher expression in footpad tumors; blue, lower expression in footpad tumors). *P* values were adjusted using the Benjamini and Hocheberg method (DESeq2). **d** Kyoto encyclopedia of genes and genomes (KEGG) pathway analysis. *P* values were obtained by using a modified fisher’s exact t test. **e** Gene set enrichment analysis (GSEA). **f** Expression of selected genes in footpad tumors relative to that in flank tumors (*n* = 3 mice). **g** Quantitative RT-PCR analysis for measuring relative mRNA levels of selected genes (*n* = 3 mice). Error bars in **f** and **g** are SEM. Source data are provided as a Source Data file.

To investigate the effect of mechanical stress on gene regulation, we compared the transcriptional profiles of tumors generated in the flank skin and the footpad. The principal components analysis of RNA sequencing (RNA-seq) data showed a separation between flank skin and footpad tumors (Fig. 7a). This pattern was confirmed by clustering based on most differently expressed genes, suggesting differences between groups and similarities within the same group (Fig. 7b). We found 1114 upregulated genes and 317 downregulated genes in footpad tumors compared to tumors of the flank skin [false discovery rate (FDR), <0.05; fold change, ≥2] (Fig. 7c). Kyoto encyclopedia of genes and genomes (KEGG) pathway analysis^44^ and gene set enrichment analysis (GSEA)^45^ revealed that genes upregulated in footpad tumors are enriched with inflammatory response genes and extracellular matrix organization genes as well as YAP signature genes (Fig. 7d, e). Significantly upregulated genes in footpad tumors include genes encoding lamins, collagens, and chemokines (Fig. 7f). Acute mechanical stress from forced wheel running further increased the transcription of the selected genes that were upregulated in footpad tumors (Fig. 7g). In order to test whether the upregulated genes in footpad tumors are enriched in human acral melanomas, we calculated gene set variation analysis (GSVA)^46^ scores using public datasets of human melanomas^47–49^. GSVA results show that the expression of the upregulated genes in mouse footpad tumors (footpad_up gene set) is higher in acral melanoma than in cutaneous melanoma (Supplementary Fig. 13a). In addition, analysis using 11,023 TCGA cases across 33 cancer types showed that the gene signature of footpad tumors positively correlates with YAP signatures in human cancers (Supplementary Fig. 13b). These results suggest that the experimental results in the mouse model have relevance with human cancer.

Higher levels of collagen accumulation were detected in the marginal region of human plantar melanoma and mouse footpad tumors (Supplementary Fig. 14). The higher YAP activity at the tumor margin is likely due to collagen accumulation and consequent extracellular matrix stiffening. B16F10 cells expressing YAP-5SA showed higher LMNA/C protein levels compared to controls (Supplementary Fig. 15a). In addition, a number of B16F10 cells implanted into the mouse footpads displayed higher levels of LMNB1 immunohistochemistry staining than cells implanted into the flank skin (Supplementary Fig. 15b). These results indicate that downregulation of lamin expression is not a major contributing factor of nuclear envelope instability associated with higher YAP activity.

To assess the importance of YAP in footpad tumor development, B16F10 cells expressing EGFP and luciferase were implanted into the footpad and the flank skin, and then verteporfin, a YAP inhibitor^50^, was administered (Supplementary Fig. 16a). Verteporfin treatment inhibited the growth of both footpad and flank skin tumors, but the effect was more pronounced in footpad tumors (Supplementary Fig. 16b). Verteporfin treatment also reduced the expression of YAP target genes in footpad tumors (Supplementary Fig. 16c). These results suggest that melanoma cells implanted into the footpad acquire a stronger dependency on YAP function.

## Discussion

This study shows that mechanical stress due to weight-bearing activity is associated with nuclear membrane instability in plantar melanoma. We observed an increase in the number of cGAS- or BAF-positive nuclear blebs and micronuclei in B16F10 cells implanted into the mouse footpad compared to cells implanted into the trunk skin. The number of cells exhibiting the DNA damage marker gamma-H2AX also increased in footpad tumors. Because weight bearing is not the only environmental factor that differs between the footpad and trunk skin, we next performed forced wheel running experiments. Application of macroscopic mechanical stress for a few hours clearly induced nuclear membrane rupture and DNA damage in melanoma cells in the mouse footpad. Interestingly, nuclear membrane rupture was frequent in the marginal region of footpad tumors, and consistent with this, cGAS-positive nuclear blebs and micronuclei were observed at a higher frequency in the margin of human plantar melanoma samples.

Micronucleus rupture is a key mechanism by which DNA structural variations occur in cancer^15–17^. Micronuclei have relatively high structural instability compared to the primary nucleus, and ruptured micronuclei are frequently observed in cultured cancer cells. Thus, it was not expected that extrinsic forces would play an important role in promoting micronuclear rupture in vivo. However, in this study, we found that physiological levels of mechanical stress significantly affected micronuclei in mouse footpad tumors. The finding is in good agreement with the fact that DNA structural variations, including chromothripsis, are frequent in human acral melanoma^1,2^. Unlike mutations caused by UV or chemical mutagens, rupture of the nuclear and micronuclear membranes not only induces DNA damage, but also triggers innate immune responses via the cGAS-STING pathway^10,18^. The expression of a group of proinflammatory genes was elevated in footpad tumors compared to tumors grown in the trunk skin. Although the cGAS-STING pathway may have apoptosis-promoting effect by inducing type I interferon signaling, recent studies have shown that promotion of cancer progression and metastasis is the dominant effect of cGAS-STING pathway activation in cancer cells^20^. Thus, weight-bearing activity is likely to promote the progression of plantar melanoma through induction of local inflammation as well as genome instability.

So, is weight-bearing exercise a risk factor for plantar acral melanoma in humans? It is important to note that nuclear membrane rupture was not induced by forced wheel running in normal tissues adjacent to footpad tumors in mice. In normal cells, the nuclear lamina protects the nuclear membrane from mechanical stress^51^. In addition, nuclear membrane proteins linked to the nuclear lamina interact with the cytoskeleton and function to resist mechanical deformations^52,53^. A recent study also showed that transient changes in the architecture of lamina-associated heterochromatin protect the nuclear envelope integrity against mechanical stress-induced damage^9^. It would be reasonable to assume that weight bearing does not act as a contributor for tumor initiation. However, unlike normal cells, cancer cells commonly contain unstable micronuclei. Furthermore, our results indicate that the nuclear envelope of melanoma cells is altered to be vulnerable to mechanical stress, and can be ruptured by mechanical stimuli applied at normal physiological levels. Therefore, mechanical stress can be a factor promoting the acquisition of malignant properties by transformed cells of primary tumors. Despite this potential risk, weight-bearing exercise should not be considered a melanoma risk factor, because the health-promoting effects of exercise will outweigh the cancer-promoting effects^54^. The fact that there was a report of an association between prostate cancer and cycling, but no clear association was found in subsequent studies, supports this thought^55^. However, in patients diagnosed with plantar acral melanoma, efforts should be made to minimize mechanical irritation of the affected sole.

YAP plays a central role in sensing and transduction of mechanical cues, particularly static mechanical stimulation such as matrix stiffness, cell geometry, and cell adhesion^31,56^. It has also been shown that stretching, i.e., dynamic mechanical stimulation, of quiescent epithelial cells cultured as confluent monolayers rapidly induces YAP activation and consequent cell cycle re-entry^38^. The in vivo mechanisms by which dynamic mechanical stimuli on a macroscopic scale directly influence cellular behavior or cancer progression have not been studied intensively and remain enigmatic^56^. In this study, we demonstrated that YAP nuclear translocation is promoted by mechanical stimulation due to physiological weight-bearing activity in mouse footpad tumors. YAP is regulated at the level of protein degradation as well as nuclear translocation^28^. Consistent with this, high levels of YAP expression were observed in the human plantar melanoma samples. The rigid extracellular matrix of cancer tissue not only increases YAP activity by itself, but also has the potential to activate YAP by more potently transmitting exogenous mechanical strain to cells. The frequent DNA damage and nuclear membrane rupture at YAP-activated sites in both mice and melanoma patients suggest that YAP affects nuclear membrane integrity. More direct evidence was acquired by cell culture experiments. LATS1/2 double knockout or YAP-5SA expression promoted nuclear membrane rupture synergistically with p53 inhibition. Although reduction in lamin expression is known to underlie nuclear membrane instability^51^, YAP-induced damage to nuclear membrane integrity occurred without lamin downregulation. We showed that pharmacological inhibition of YAP reduced the proliferation of melanoma cells in the mouse footpad. However, because YAP plays a variety of roles in cancer, it is difficult to assess the contribution of YAP-induced nuclear membrane rupture to cancer development.

We speculate that the mechanical stress from weight-bearing activity in plantar acral melanoma acts in two stages (Supplementary Fig. 16d). First, by stimulating the nuclear translocation and stabilization of YAP, it weakens the integrity of the nuclear envelope in cells undergoing tumorigenesis. Loss of RB and TP53, which is a prevalent event in human cancers, may be a prerequisite or a contributing factor for significant loss of nuclear envelope integrity. Further studies are needed to investigate how YAP activation and tumor suppressor loss weaken the system that maintains nuclear membrane integrity. In the second stage, weakened nuclear and micronuclear membranes are damaged by sustained mechanical stress transmitted to the cancer cells. It is likely that nuclear membrane rupture is frequent in the tumor margin because the activity of YAP is particularly high at the area. Previous studies have shown that YAP nuclear import is promoted by stretching of nuclear pores, as a result of either actomyosin contractility or of direct application of force to the nucleus^57,58^. Thus, it is possible that YAP activation is not only a driver of nuclear membrane instability, but also a consequence of mechanically induced nuclear transformation. Although not presented in this study, direct evidence that mechanical stress is higher in the margin of plantar melanoma will support this possibility. An increase in matrix stiffness due to collagen accumulation may be a reason for the high YAP activity in the marginal region. Although melanoma cells express collagen genes at a high level, infiltrated fibroblasts can increase the activity of YAP by accumulating extracellular matrix components. It is also possible that fibroblasts add mechanical stress to cancer cells by exerting tension on the extracellular matrix.

Previous studies have focused on mechanical stress caused by the migration of cancer cells as the main cause of nuclear envelope rupture^13,14^. Our study shows that mechanical stress from weight-bearing activity can damage the nuclear membrane of primary acral melanoma. Our findings provide an explanation for the intriguing clinical observation that plantar acral melanoma occurs mainly in areas with high mechanical stress on the sole of the foot. Nuclear membrane rupture is a mutational process of melanoma that is not related to ultraviolet radiation, and also reveals a mechanism by which YAP contributes to cancer development. In this study, we developed a simple but effective mouse model for assessing the effect of macroscopic mechanical stimulation on cancer cell behaviors. However, there may be other factors induced by excessive exercise such as increased metabolic activity and plantar edema that may contribute to the observed phenotypes, so it is necessary to validate the results with other experimental models. Acral melanomas form not only on the soles of the feet, but also on the palms or under the nails. It will be interesting to study whether pressure stress plays an important role in melanoma generated in these areas and also induces nuclear membrane rupture. Also, further studies are needed to determine whether nuclear membrane rupture in melanoma contributes to malignancy acquisition and responsiveness to immune checkpoint inhibitor therapy, and whether nuclear membrane instability is seen in other cancers in which YAP is highly activated.

## Methods

### Study approval

Experiments on human samples were reviewed and approved by the Institutional Review Board of Severance Hospital (2020-2401-002). Only essential information of participants collected with prior consent is disclosed. There was no compensation for participants. KAIST IACUC approved the animal care and experimental procedures used in this study (KA2017-26).

### Cell implantation in mice

All mice were housed in an approved animal facility at KAIST under a 12 h light-dark cycle and 50 ± 10% humidity and a temperature of 22 ± 2 °C. Mice were allowed ad libitum access to a standard diet (PMI LabDiet) and water. For syngeneic graft experiments, B16F10 mouse melanoma cells (1 × 10^6^) were simultaneously injected into the flank, tongue, and the hind footpad of female C57BL/6J mice (6 weeks old). For xenograft experiments, RPE1-LATS1/2 dKO cells (1 × 10^6^) were injected into 6-week-old female *Foxn1*-null (nude) mice. Tumor formation and volume were monitored every 1 or 2 days for the duration of the experiment. All mice were euthanized before the maximum allowable tumor size (20 mm in diameter) was exceeded.

### Forced wheel running

Custom-designed electronic device was used to force mice to run on the wheel (diameter: 13 cm). Mice with a footpad tumor were placed on a soft latex mattress (density: 50 kg/m^3^) for 24 h before wheel running. The mattress was replaced when it was damaged by mice during the 24 h period. Multiple cycles of forced wheel running were performed to apply mechanical stress on the mouse footpad. In each cycle, the mouse ran steadily with the wheel speed at 0.05 m/s for 1 h. The average running distance of one exercise cycle was 210 m, and a 15 min break was given with drinking water between running cycles to prevent the exhaustion of mice.

### In vitro compressive load

Cells were cultured on 35 mm petri dishes with a 14 mm glass-bottom microwell (MatTek). To apply a compressive force, a weight (5 g or 10 g) was placed on a circular coverslip (8 mm diameter, Marienfeld Superior) mounted on the cells. The approximate force per cell was calculated considering the area of the coverslip and the number of cells under the coverslip: Force/cell = {[weight (kg) × 9.8 (m/s^2^)/area of coverslip (mm^2^)] × total area of cells under coverslip (mm^2^)}/total number of cells under coverslip.

### Cell culture and reagents

Cells were acquired from American Type Culture Collection (ATCC) or Korean Cell Line Bank (KCLB) and large frozen stocks were made to ensure against contaminations by other cell lines. All cell lines were used within five passages after reviving from the frozen stocks. Cells were free of mycoplasma contamination as determined by staining cells with DAPI. Cells were cultured in the following media: [DMEM (Welgene) for B16F10 (ATCC: CRL-6475™) and A375SM (KCLB: 80004); DMEM/F12 (Welgene) for RPE1 (ATCC: CRL-4000™); MEM (Welgene) for SKMEL28 (ATCC: HTB-72™) and RPMI-7951 (ATCC: HTB-66™)] supplemented with 10% FBS and 1% penicillin/streptomycin. Stable clonal cell lines were generated as described previously^40^ using the following constructs: YAP 5SA (Addgene 27371), NLS-EGFP (Addgene 17300); H2B-mCherry (Addgene 20972), EGFP-cGAS (Addgene 127661), and TK-GFP-LUC (gift from Mi-Young Kim, KAIST). In brief, transfected cells were treated with selection antibiotics or sorted based on EGFP fluorescence. Afterwards the cell line was cloned by liming dilution, and single clones were expanded individually and selected for experiments. RPE1 cell lines carrying inactivating mutations in TP53 genes were established using the CRISPR-Cas9 system according to the previously published protocol^27^. Briefly, TP53 sgRNA was cloned into pSpCas9(BB)-2APuro vector (Addgene 62988) and transfected to RPE1 cells. After transfection, cells were selected with puromycin, and single clones were expanded individually. The sgRNA sequence targeting TP53 was as follows: 5′-CACCCGCGTCCGCGCCA-3′. Cytochalasin D (Sigma-Aldrich) was used at 200 nM. LPA (Sigma-Aldrich) was used at 20 μM, added at 30 min before fixation. All cell lines were cultured under humidified conditions at 37 °C and 5% CO_2_.

### Plasmid and siRNA transfection and lentiviral infection

Plasmids and siRNAs were transfected with Lipofectamine LTX and PLUS reagent (Invitrogen) and Lipofectamine RNAiMAX (Invitrogen), respectively, according to the manufacturer’s protocol. Scramble siRNAs (Qiagen) were used as a negative control. The lentiviral transfer vector DNA, together with psPAX2 packaging and pMD2.G envelope plasmid DNAs, were co-transfected to HEK293T cells (ATCC: CRL-3216™). Cell culture supernatant was filtered and used to infect cells.

### Transwell migration assay

1:4 mixture of growth factor reduced matrigel and serum-free media was prepared, and transwell insert for 24-well plate (SPL) was coated with 50 μl/insert of the mixture. Cells (10^5^ cells/insert) were suspended in serum-depleted media, and were seeded upon matrigel coated insert. The insert was placed on 24-well plate, and media supplemented with 10% FBS was added under transwell insert as chemo-attractants. EGFP and DAPI fluorescence was imaged in cells that passed through the transwell after the removal of non-migrated cells.

### Live cell imaging

Live imaging of RPE1 cells stably expressing H2B-mCherry was performed with 35 mm petri dishes with a 14 mm glass-bottom microwell (MatTek) on a DeltaVision Spectris Imaging System (Applied Precision) equipped with an environmental chamber.

### Cell viability assay

Cells were seeded at 2500 cells/well in 96-well microplates (SPL). Cells were incubated for the indicated time and viable cells were quantified by 450 nm absorbance measurement 1 h after treatment of Cell Counting Kit-8 reagent (CCK8; Dojindo) according to the manufacturer’s protocol.

### Immunofluorescence staining of culture cells and frozen tissues

Cells were fixed with 4% paraformaldehyde (PFA) for 8 min at room temperature (RT). After fixation, 0.1% Triton X-100 (Sigma-Aldrich) was applied for permeabilization. Cells were incubated with primary antibodies for 1 h at RT. Cells were incubated with Alexa Fluor 488- or 594-conjugated anti-mouse or anti-rabbit secondary antibodies (Invitrogen; 1:1000 dilution) for 1 h at RT. For staining actin filaments, Alexa Fluor 488- or 594-conjugated phalloidin (Invitrogen) was used according to the manufacturer’s protocol. For immunofluorescence staining of the mouse tumor, tumor tissues were fixed in 4% PFA overnight in 4 °C, dehydrated in 30% sucrose solution, and embedded in tissue freezing medium (Leica). Cryosections were blocked with 3% donkey serum in PBST (0.3% Triton X-100 in PBS) and then incubated at 4 °C overnight with primary antibodies. The samples were washed five times with PBS, followed by incubation with secondary antibodies (Invitrogen; 1:1000 dilution) for 2 h at 4 °C. Fluorescence images were acquired using a DeltaVision Spectris Imaging System (Applied Precision) and LSM800 confocal microscope (Carl Zeiss).

### Immunofluorescence staining of paraffin sections

Melanoma paraffin blocks (13 plantar and 13 cutaneous melanomas) were retrieved from the Department of Dermatology of Severance Hospital, South Korea. After deparaffinization and heat-induced antigen retrieval, samples were blocked with 3% donkey serum in PBST (0.3% Triton X-100 in PBS) and then incubated at 4 °C overnight with primary antibodies. After several washes, the samples were incubated for 2 h at 4 °C with Alexa Fluor 488- or 594-conjugated secondary antibodies (Invitrogen; 1:1000 dilution). To eliminate the autofluorescence, quenching agent (TrueVIEW, Vector Laboratories) was used according to manufacturer’s instructions. Sections were mounted with anti-fade mounting medium (Vibrance, Vector Laboratories) and immunofluorescent images were acquired using an LSM800 confocal microscope (Carl Zeiss).

### Immunohistochemistry

Paraffin sections were deparaffinized and antigen-retrieved using a pressure cooker. Sodium-citrate buffer (pH 6) or Tris-EDTA buffer (pH 9) were used as an antigen retrieval solution, depending on the type of the antigen. The Sections were incubated with BLOXALL (Vector Laboratories) at RT, blocked with 3% horse (or goat) serum in PBS or PBST (0.3% Triton X-100 in PBS), and then incubated at 4 °C overnight with primary antibodies. The samples were washed and incubated with biotinylated IgG anti-mouse or anti-rabbit secondary antibody (Vector Laboratories; 1:200 dilution) for 30 min at RT. VECTASTAIN ABC kit and DAB was used according to the manufacturer’s protocol (Vector Laboratories). Sections were counterstained with Harris hematoxylin (Papanicolaou solution 1a, Merck) and mounted with mounting medium (DAKO). For hematoxylin and eosin slides, sections were stained with hematoxylin and eosin (BBC biochemical) according to the manufacturer’s protocol. For histological assessment of collagen deposition, trichrome staining was performed using a Masson Trichrome Staining Kit (Sigma-Aldrich). Immunohistochemistry images were acquired using a Pannoramic SCAN II (3DHISTECH) and analyzed by CaseViewer 2.2 (3DHISTECH).

### Image analysis

To quantify YAP localization (Fig. 5b and Supplementary Fig. 11a) and NLS-EGFP release from the nucleus (Fig. 5d), we established a customized pipeline by using CellProfiler software (Broad Institute). The nuclei were identified as a primary object based on DAPI staining (threshold strategy = Global; Thresholding method = Otsu), and perinuclear cytoplasmic areas were identified by expanding the primary object (method = Distance-N; Expansion = 9 pixels). Nuclear/cytoplasmic (N/C) ratios of YAP mean fluorescence intensities were determined to define YAP distribution patterns either as nuclear (Nuc: N/C ratio > 1.2) or cytoplasmic (Cyto: N/C ratio < 0.9) or equivalent distribution in the nucleus and cytoplasm (Nuc/Cyto: 0.9 ≤ N/C ratio ≤ 1.2). N/C ratios of EGFP mean fluorescence intensity of less than 1.5 were considered indicative of cytoplasmic NLS-EGFP release. The shape of the cell nucleus in tumor sections and cultured cells was also quantified using CellProfiler (Supplementary Figs. 1b, 2a, 10c). Compactness measures irregularity of an object. The compactness of a filled circle is 1.0, and irregular objects have a value greater than 1.0. A circularity value of 1.0 represents a perfect circle. As the object is elongated, the circularity becomes smaller than 1.0. For quantification of immunofluorescence staining, multiple sections from at least three independent experiments/mice/human melanomas were examined. When the distinction between micronucleus and nuclear bleb was ambiguous, they were counted without distinction and the results were shown on a single graph. Quantification of gamma-H2AX, cGAS, BAF, and YAP immunostaining in tissue sections was based on qualitative (yes/no) measurement by two researchers with much experience performing immunostaining image analysis, and human samples were analyzed blindly.

### Immunoblotting

For phosphorylated protein detection, membranes were blocked with 2% BSA in PBS. Membranes were incubated with primary antibodies in TBST buffer with 5% BSA or skim milk. After incubation, membranes were washed with TBST buffer, and incubated with HRP-linked anti-mouse IgG or anti-rabbit IgG secondary antibodies (1:2000 dilution) at 4 °C for 1 h. Target proteins were detected using enhanced chemiluminescence western blot detection solution (LumiGlo, KPL; Western Bright, Advansta). For quantification of immunoblot band intensity, the integrated intensity of inverted images were measured using image analysis software (ImageJ).

### Electron microscope imaging

The cell nuclei of mouse footpad tumors were examined with a transmission electron microscope (Tecnai G2 Spirit Twin, FEI) after routine fixation, embedding, and sectioning. Briefly, samples were fixed for 24 h at 4 °C. Samples were washed with PBS, and postfixed in 1% osmium tetroxide (Electron Microscopy Sciences). Samples were treated with propylene oxide, and infiltrated by a mixture of propylene oxide and EMBed 812 resin (EMS). Samples were embedded in 100% EMBed 812 resin and polymerized at 60 °C for 24 h. Ultrathin (70 nm) sections were prepared using an ultramicrotome (Leica, EM UC7) and collected on 200-mesh formvar/carbon-coated nickel grids. Images were acquired using a Capture Engine software (AMT Imaging).

### In vivo bioluminescence imaging

B16F10 cell transfected with TK-GFP-LUC reporter plasmid were implanted subcutaneously into the footpad region and flank skin region of 6 week-old *Foxn1*-null mice. After 7 days, 2 mg of verteporfin (Sigma) diluted in 100 μl PBS was injected intraperitoneally every 2 days. Bioluminescence signal from mice was measured with IVIS lumina xenogen (Caliperls) 12 min after D-luciferin (Goldbio, 115144-35-9) was injected into mice retro-orbitally at 10 µg/g body weight. The following conditions were used for image acquisition: exposure time = auto exposure, binning = 60, field of view = 5 × 5 cm, and f/stop = 5. The bioluminescence images were analyzed by Living Image 2.60 software (PerkinElmer) specialized for IVIS system.

### Quantitative RT-PCR (qRT-PCR)

Total RNA was extracted from cells (RNeasy kit, Qiagen). A total of 1 μg of extracted RNA was annealed with oligo dT primer (Roche Life Science), and reverse-transcribed to cDNA using M-MLV reverse transcriptase (Promega) in the presence of RNase Inhibitor (RNasin Plus, Promega). cDNA was mixed with primers and iQ SYBR Green Supermix (Bio-Rad), and mRNA expression levels were measured by real-time qRT-PCR (CFX96 system, Bio-Rad).

### RNA sequencing

B16F10 tumor cells from primary implantation sites were isolated. RNAs were extracted by RNeasy Kit (Qiagen) according to the manufacturers’ protocol. cDNA library was constructed using TruSeq Stranded Total RNA LT Sample Prep Kit (Gold) according to the manufacturer’s protocol. Raw sequencing data were quality-checked by FastQC and filtered using Trimmomatic (version 0.36) tool. The trimmed data were subsequently aligned to mouse reference genome (mm10) using HISAT2 (ver.2.1.0) The read count data were filtered, resulting in remaining transcripts with gene IDs,read counts, FPKM (Fragments Per Kilobase of transcript per Million Mapped reads) and TPM (Transcripts Per Kilobase Million). The filtered read count data were next normalized by DEseq2 package, and variance stabilizing transformation were applied to the count data. Gene ontology annotation analysis in upregulated and downregulated genes was performed using DAVID software with GOTERM_BP_DIRECT database. The gene set enrichment analysis (GSEA; Broad Institute) was performed using C1 hallmark gene sets, C2 curated gene sets, C3 regulatory target gene sets, and C6 oncogenic gene sets (MSigDB). For calculating gene set variation analysis (GSVA) scores, publicly available datasets regarding human melanoma gene expression were used. For each dataset, metastatic melanomas in lymph nodes were filtered out. Melanomas were categorized as either acral or cutaneous according to their primary sites. Count matrices of processed dataset were combined and a batch effect was adjusted using ComBat-Seq^59^. After batch correction, the expression data underwent variance-stabilizing transformation using DESeq2, and the output was used to calculate GSVA score. When running the GSVA algorithm, the kernel function was set as Gaussian.

### Genome analysis

Genomic DNA materials were extracted from fresh frozen tumor tissues and original cell line by using DNeasy Blood and Tissue kits (QIAGEN). DNA libraries for WGS were generated by a TruSeq PCR-Free Library Preparation Kit (Illumina) from 1 μg of genomic DNA materials. WGS was performed on the Illumina platform to generate a minimal read depth of 90X for samples. Short-reads were aligned to a mouse and human concatenated reference, and reads mapped to human reference genome (GRCh37) were extracted to remove mouse contaminated reads using BWA-MEM software^60^. Somatic single-nucleotide substitutions and indels were called by Mutect2, and Strelka. The call set was combined together and filtered using in-house scripts. After filtering the call set, mutations present in only one sample were used in the final analysis. Genomic rearrangements were called using Delly, and filtered using in-house scripts as previously described^61^. Structural variations were further inspected using IGV. After filtering the structural variation call set, breakpoints present in only one sample were used in the final analysis. The circos plots were created using Circlize package in R.

### Statistical analysis

Unless otherwise noted, all experimental results were taken from at least three independent experiments. Data are presented as the mean ± SEM, unless otherwise noted. The numeric data was normalized by dividing all values of control and treatment groups by the mean of the control. Student’s t tests or one-way ANOVA with post hoc tests were used for calculating statistical significance. In multiple comparisons, the significance level was adjusted using Bonferroni corrections. Data analysis was performed using GraphPad Prism version 7 (GraphPad Software), and statistical significance was considered when *p*-value was <0.05. Error bars of the graph represent the standard error of the mean.

### Reporting summary

Further information on research design is available in the Nature Research Reporting Summary linked to this article.

## Supplementary information

Supplementary Information

Reporting summary

## Data Availability

RNA-seq data generated in this study have been deposited in the Gene Expression Omnibus (GEO): GSE192835. Deep DNA sequencing data generated in this study have been deposited in the NCBI Sequence Read Archive (SRA): PRJNA799072. All other data are available in the main text or in the supplementary materials. Public datasets were obtained from publicly accessible repositories: bioRxiv (10.1101/2020.11.14.383083), dbGap phs001486, TCGA Research Network (https://gdc.cancer.gov/about-data/publications/pancanatlas). The genomic coordinates of human and mice were obtained from publicly accessible repositories: GRCh37 (https://www.ncbi.nlm.nih.gov/assembly/GCF_000001405.13/); mm10 (https://www.ncbi.nlm.nih.gov/assembly/GCF_000001635.20/). Source data are provided with this paper.

## Source data

Source Data
