# Publisher Correction: Weight-bearing activity impairs nuclear membrane and genome integrity via YAP activation in plantar melanoma

**DOI:** 10.1038/s41467-022-30476-4

**Published:** 2022-05-10

**Authors:** Jimyung Seo, HyunSeok Kim, Kyoung Il Min, Changgon Kim, Yongsoo Kwon, Zhenlong Zheng, Yusung Kim, Hyung-Soon Park, Young Seok Ju, Mi Ryung Roh, Kee Yang Chung, Joon Kim

**Affiliations:** 1 Graduate School of Medical Science and Engineering, Korea Advanced Institute of Science and Technology (KAIST), Daejeon, Korea; 2 Department of Dermatology, Severance Hospital, Cutaneous Biology Research Institute, Yonsei University College of Medicine, Seoul, Korea; 3 Department of Mechanical Engineering, Korea Advanced Institute of Science and Technology (KAIST), Daejeon, Korea; 4 Department of Dermatology, Gangnam Severance Hospital, Cutaneous Biology Research Institute, Yonsei University College of Medicine, Seoul, Korea

**Keywords:** Melanoma, Melanoma, Cell signalling, Genomic instability

Correction to: *Nature Communications* 10.1038/s41467-022-29925-x, published online 25 April 2022.

The original version of the Supplementary Information associated with this Article included an incorrect Supplementary Data 1 file, which was an incorrect version of the Source Data. The original Source Data file is correct. The HTML has been updated to remove Supplementary Data 1.

