# Supplementary material for: Weight-bearing activity impairs nuclear membrane and genome integrity via YAP activation in plantar melanoma
Source: Nat Commun. 2022 Apr 25;13:2214. doi: 10.1038/s41467-022-29925-x (PMC9038926; doi:10.1038/s41467-022-29925-x)
Supplement: Supplementary file 1 — Supplementary Information [file 41467_2022_29925_MOESM1_ESM.pdf]

SUPPLEMENTARY MATERIALS

Supplementary table 1: Melanoma sample information. In this study, plantar and cutaneous melanoma nodules resected from 26 patients (Severance Hospital, Seoul, Korea) were analyzed.

Case No.	Site	Category	Clark level	Mitotic rate (n/10 HPFs)	Tumor depth (mm)	AJCC Melanoma Staging	Tumor cell type
1	Sole and toe	Plantar	IV	13	5	IIC	Spindle
2	Heel	Plantar	IV	7	5	IIC	Epithelioid
3	Sole	Plantar	IV	2	5	IIC	Epithelioid and spindle
4	Heel	Plantar	V	1	4.8	IIB	Epithelioid and spindle
5	Sole	Plantar	V	1	4.5	IIB	Epithelioid
6	Heel	Plantar	IV	13	4.2	IIC	Nevus-like and small cell
7	Heel	Plantar	IV	26	4.1	IIC	Nevus-like
8	Sole	Plantar	IV	12	4	IIB	Epithelioid
9	Heel	Plantar	IV	11	3.5	IIB	Epihtelioid
10	Heel	Plantar	IV	0	3	IIA	Spindle
11	Sole	Plantar	V	45	7	IIB	Epithelioid
12	Heel	Plantar	IV	1	2.1	IB	Epithelioid
13	Sole	Plantar	IV	1.4	2.2	IIA	Epithelioid and dendritic
14	Cheek	Cutaneous	V	12	6	IIC	Epithelioid
15	Thigh	Cutaneous	IV	6	6	IIB	Epithelioid and spindle
16	Chest	Cutaneous	IV	2	5	IIB	Epithelioid
17	Neck	Cutaneous	IV	2	4.1	IIB	Epithelioid
18	Thigh	Cutaneous	IV	5	3	IIB	Epithelioid
19	Ear	Cutaneous	IV	0.6	2.5	IIB	Epithelioid
20	Cheek	Cutaneous	V	24	3.2	IIA	Epithelioid
21	Scalp	Cutaneous	IV	19	2.5	IB	Spindle and nevus-like
22	Back	Cutaneous	IV	10	2.1	IIB	Nevus-like
23	Thigh	Cutaneous	IV	5	3	IIB	Epithelioid
24	Flank	Cutaneous	IV	17	3	IIA	Epithelioid
25	Flank	Cutaneous	IV	10	3.2	IIb	Epithelioid
26	Back	Cutaneous	IV	6	2	IIA	Nevus-like

Supplementary table 2: Target sequences of siRNA, qRT-PCR primers, and primary antibodies used in this study.

siRNA target sequences	
**Target**	**siRNA sequence (5′ to 3′)**
Control	Allstars negative control siRNA (Qiagen)
INCENP	AGA UCA ACC CAG AUA ACU A
LMNA	CUG GAC UUC CAG AAG AAC A
LMNB1	CGC GCU UGG UAG AGG UGG AUU
TAZ	CCA UGA GCA CAG AUA UGA GAU
TP53	GAC UCC AGU GGU AAU CUA C
YAP	CGG UUG AAA CAA CAG GAA UUA

qRT-PCR primers			
**Gene**	**Species**	**Primer**	**Primer sequence**
Acta2	Mouse	Forward	GCTGGTGATGATGCTCCCA
		Reverse	GCCCATTCCAACCATTACTCC
Ankrd1	Mouse	Forward	GCTGGTAACAGGCAAAAAGAAC
		Reverse	CCTCTCGCAGTTTCTCGCT
Col12a1	Mouse	Forward	TGGTTAACGAACTGGGTCGG
		Reverse	GAAAAGCATCAGGCGGACAC
Ctgf	Mouse	Forward	GGGCCTCTTCTGCGATTTC
		Reverse	ATCCAGGCAAGTGCATTGGTA
Cyr61	Mouse	Forward	CTGCGCTAAACAACTCAACGA
		Reverse	GCAGATCCCTTTCAGAGCGG
Gapdh	Mouse	Forward	AAGGTCATCCCAGAGCTGAA
		Reverse	CTGCTTCACCACCTTCTTGA
Itga5	Mouse	Forward	ATATCTGCCAGCGCATCTC
		Reverse	AGGCATTGAGGCAGAAGCTA
Lmnb1	Mouse	Forward	CAACTGACCTCATCTGGAAGAC
		Reverse	TAAGACTGTGCTTCTCTGAGC
Lmna	Mouse	Forward	TCCAATGTGCGCTTCTCAC
		Reverse	AAGACCCTTGATTCTGTGGC
Tead4	Mouse	Forward	TGATGCAGAGGGTGTATGGA
		Reverse	GATCAGCTCATTCCGACCAT
GAPDH	Human	Forward	CAACGGATTTGGTCGTATTGG
		Reverse	GCAACAATATCCACTTTACCAGAGTTAA
INCENP	Human	Forward	CACAACTACGGGATGGATCTGA
		Reverse	GATATCCTCCAAGTCCAGTGGG

Immunoblotting				
**Antibody**	**Source**	**Manufacturer**	**Catalog Number**	**Dilution**
c-Myc	Rabbit	Abcam	ab32072	1:1000
FLAG	Mouse	Sigma-Aldrich	F1804	1:1000
gamma-H2AX	Rabbit	Cell Signaling Technology	2577	1:1000
GAPDH	Mouse	Santa Cruz Biotechnology	sc59540	1:2000
Lamin A/C	Rabbit	Cell Signaling Technology	2032	1:1000
Lamin B1	Rabbit	Abcam	ab16048	1:3000
LATS1	Rabbit	Cell Signaling Technology	3477	1:1000
LATS2	Rabbit	Cell Signaling Technology	5888	1:1000

P53	Mouse	Santa Cruz Biotechnology	sc126	1:5000
phospho-TAZ	Rabbit	Cell Signaling Technology	59971	1:1000
phospho-YAP (Ser127)	Rabbit	Cell Signaling Technology	4911	1:1000
TAZ	Rabbit	Cell Signaling Technology	83669	1:1000
YAP	Mouse	Santa Cruz Biotechnology	sc101199	1:1000
**Immunofluorescence**				
**Antibody**	**Source**	**Manufacturer**	**Catalog Number**	**Dilution**
Alexa Fluor™ 488 Phalloidin		Invitrogen	A12379	1:1000
Alexa Fluor™ 594 Phalloidin		Invitrogen	A12381	1:1000
BAF	Rabbit	Abcam	ab129184	1:100
cGAS	Rabbit	Cell Signaling Technology	15102	1:100
cGAS (mouse specific)	Rabbit	Cell Signaling Technology	31659	1:250 or 1:125
FLAG	Mouse	Sigma-Aldrich	F1804	1:2000
FLAG	Rabbit	Sigma-Aldrich	F7425	1:2000
gamma-H2AX	Rabbit	Cell Signaling Technology	2577	1:800 or 1:400
Lamin A/C	Mouse	Santa Cruz Biotechnology	sc7292	1:1000
Lamin B1	Rabbit	Abcam	ab16048	1:1000 or 1:500
YAP	Mouse	Santa Cruz Biotechnology	sc101199	1:500 or 1:250
YAP	Rabbit	Cell Signaling Technology	14074	1:100
**Immunohistochemistry**				
**Antibody**	**Source**	**Manufacturer**	**Catalog Number**	**Dilution**
gamma-H2AX	Rabbit	Cell Signaling Technology	2577	1:100
Lamin B1	Rabbit	Abcam	ab16048	1:100
Myc	Rabbit	Millipore	06-340	1:100
YAP	Mouse	Santa Cruz Biotechnology	sc101199	1:100
YAP	Rabbit	Cell Signaling Technology	14074	1:100

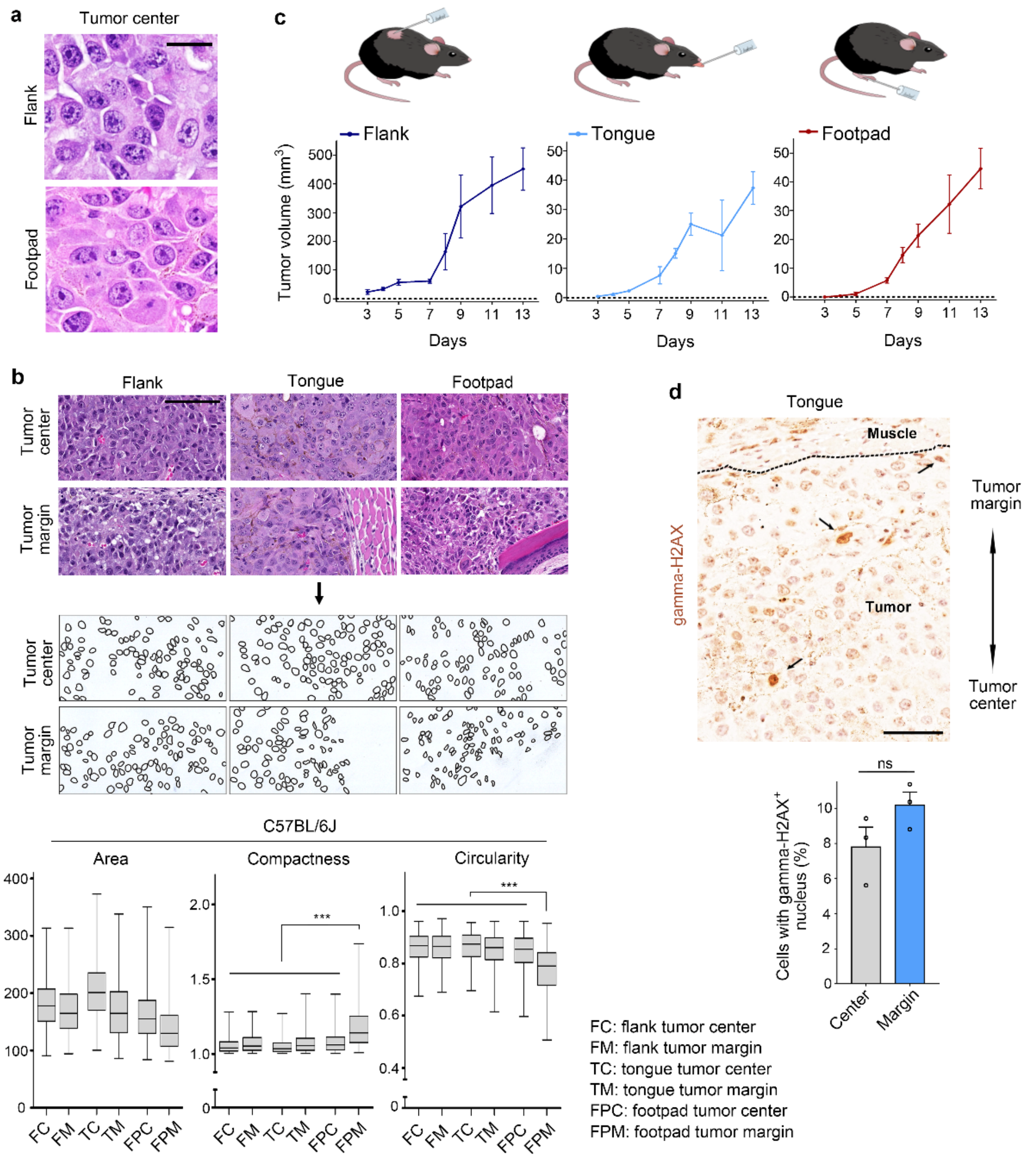

Supplementary figure 1: Nuclear morphology and growth rate of tumors generated by implantation of B16F10 cells. a, Nuclear morphology at the tumor center. B16F10 cells were simultaneously implanted into the flank and the footpad of C56BL/6J mice. **b**, Quantification of nuclear morphology using an image analysis software (CellProfiler). Data were analyzed by one-way ANOVA with Tukey's post hoc test (n=3 mice; ***P<0.0001). Box plots indicate median (middle line), 25th and 75th percentile (box), and 1st and 99th percentile (whiskers). Each data represents a result obtained from individual cells. The number of cells analyzed in each box: (FC) 765, (FM) 637, (TC) 681, (TM) 599, (FPC) 715 and (FPM) 703. Comparison of tumor growth rate by the implantation site. C57BL/6J mice were implanted with the same number of B16F10 cells at 3 sites and followed for 13 days (n=3 mice). **d**, Detection and quantification of gamma-H2AX^{+} cells (arrows) in a tongue tumor generated by implantation of B16F10 cells. Error bars are SEM (n=3 mice; two-tailed unpaired t test; ns, not significant). Each dot represents data obtained from one mouse. Size bars: 20 µm (a), 100 µm (b), and 50 µm (d). Source data are provided as a Source Data file.

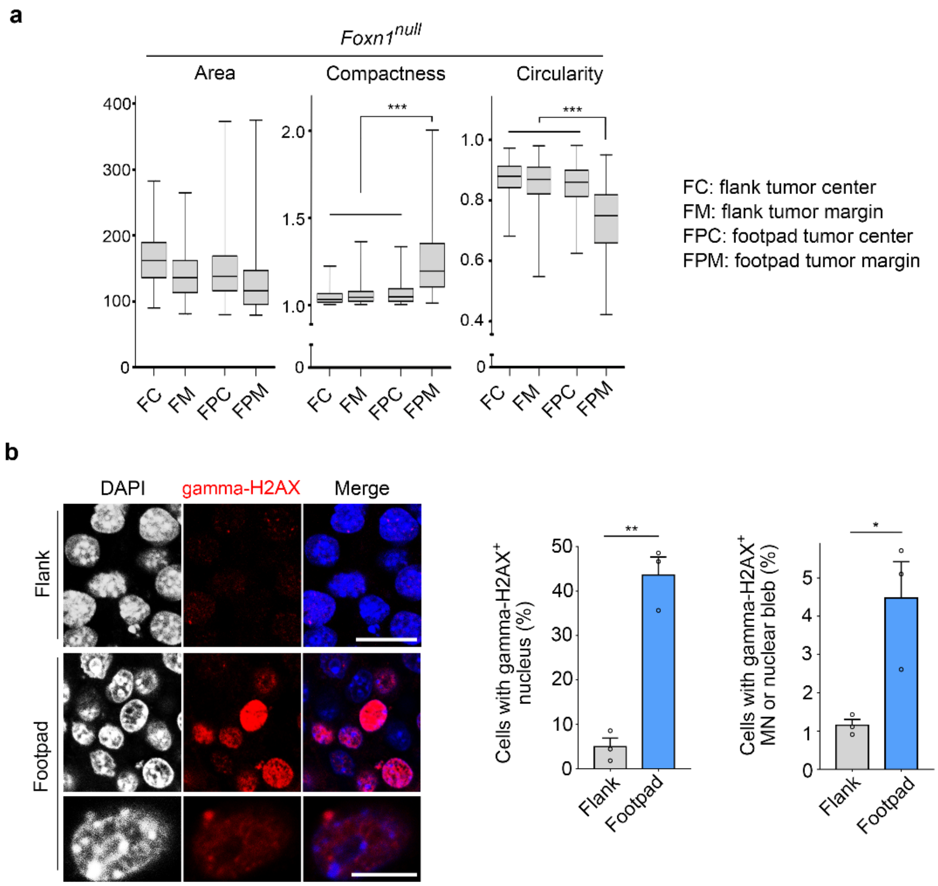

Supplementary figure 2: Nuclear morphology and DNA damage of tumor cells in *Foxn1*-null mice. a, Quantification of the nuclear morphology. B16F10 cells were simultaneously implanted into the flank and the footpad of *Foxn1*-null (nude) mice. Data were analyzed by one-way ANOVA with Tukey's post hoc test (n=3 mice; ***P<0.0001). Box plots indicate median (middle line), 25th and 75th percentile (box), and 1st and 99th percentile (whiskers). Each data represents a result obtained from individual cells. The number of cells analyzed in each box: (FC) 761, (FM) 656, (FPC) 708 and (FPM) 562. **b**, Labeling of tumor sections with gamma-H2AX antibody, and quantification of gamma-H2AX$^+$ nuclei and gamma-H2AX$^+$ MN/nuclear blebs. Error bars are SEM (n=3 mice; **P=0.001 and *P=0.0259; two-tailed unpaired t test). Each dot represents data obtained from one mouse. Size bars: 20 μm (b, top panel) and 10 μm (b, bottom panel). Source data are provided as a Source Data file.

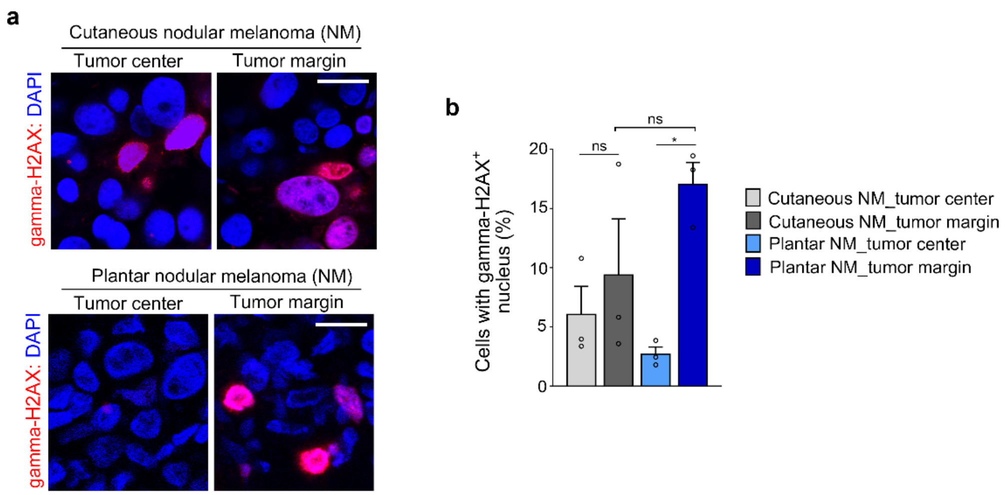

Supplementary figure 3: DNA damage of tumor cells in human melanomas. a, Detection of gamma-H2AX^{+} cells in human nodular melanomas resected from the cheek (cutaneous) and the heel of the foot (plantar). Representative images of three independent experiments are shown. **b**, Quantification of nuclear gamma-H2AX staining. Error bars are SEM (n=3 human melanomas per group; *P=0.0289; One-way ANOVA with Tukey's post hoc test). Each dot represents data obtained from one patient. Size bars: 10 µm (a). Source data are provided as a Source Data file.

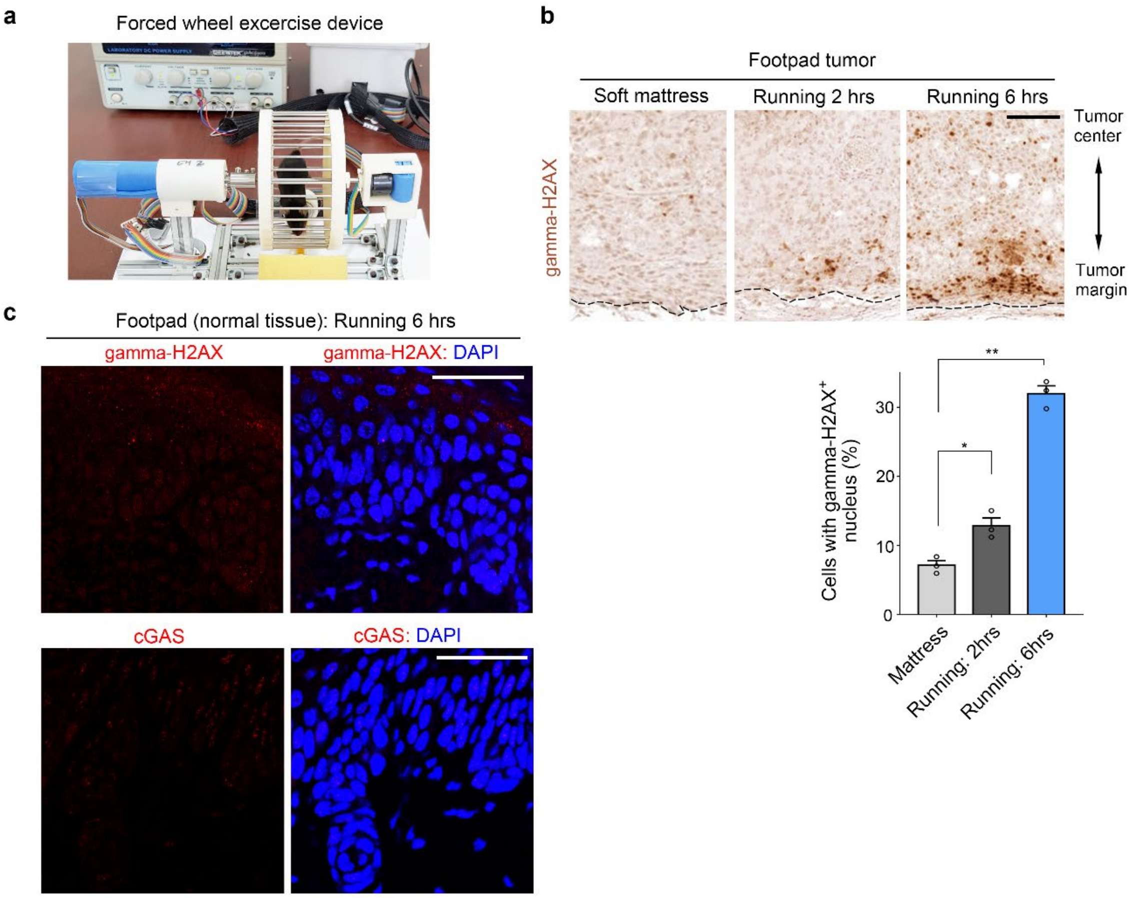

Supplementary figure 4: Forced wheel running to increase mechanical stress on the mouse footpad. **a**, The homemade forced wheel running device used in this study. **b**, Detection and quantification of gamma-H2AX$^+$ cells in mouse footpad tumors after 2 or 6 hrs of forced wheel running. Error bars are SEM (n=3 mice per group; *P=0.0129 and **P<0.0001; two-tailed unpaired t test). Each dot represents data obtained from one mouse. **c**, Gamma-H2AX and cGAS immunofluorescence staining in normal footpad tissue adjacent to the tumor after 6 hrs of forced wheel running. Representative images of three independent experiments are shown. Size bars: 100 μm (b) and 40 μm (c). Source data are provided as a Source Data file.

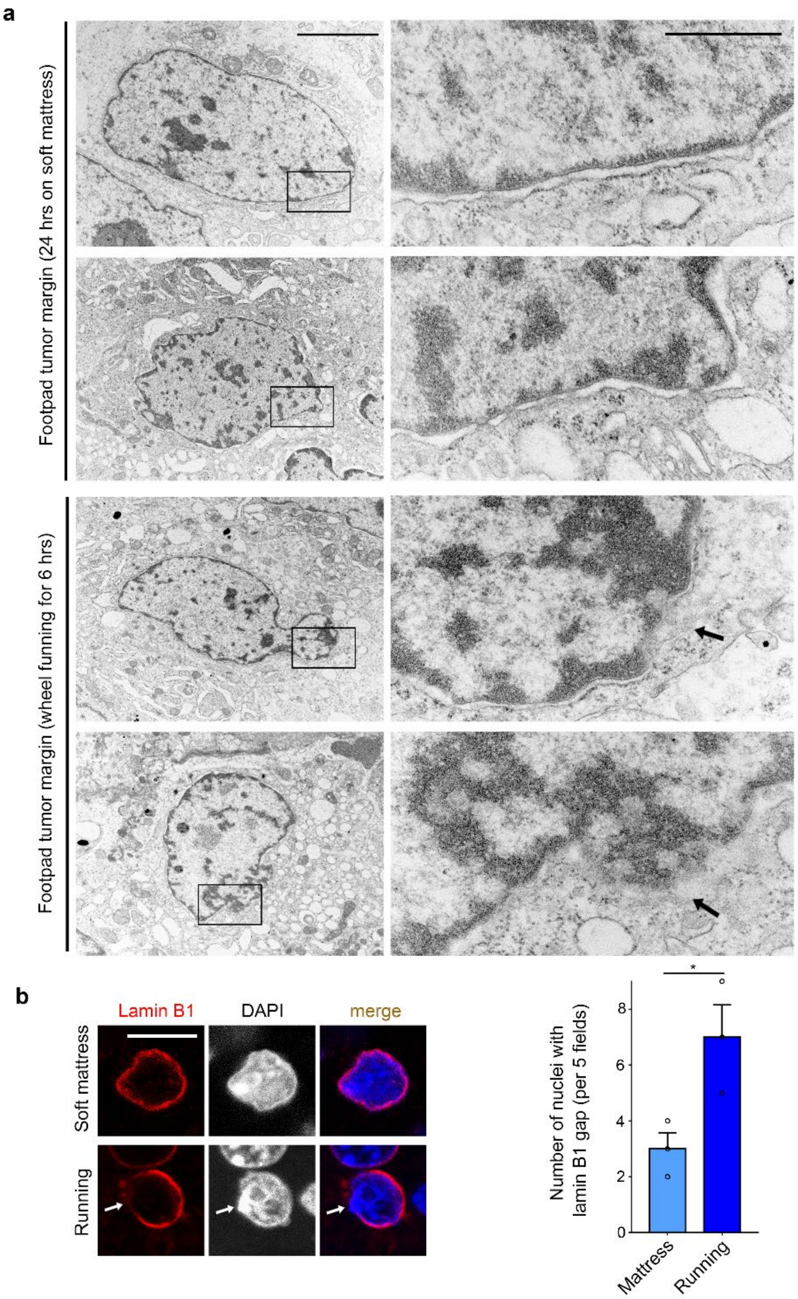

Supplementary figure 5: Electron microscopy and immunofluorescence analysis showing nuclear envelope damage after wheel running. a, Transmission electron micrographs. Mice with footpad tumors were placed on a soft mattress for 24 hrs. Then mice were forced to run on the wheel for a total of 6 hrs before analysis. The images shown are representative analysis results of three mice per group. **b**, Detection and quantification of cells showing nuclear Lamin B1 gap. Arrows indicate nuclear envelope rupture. Error bars are SEM (n=3 mice per group; *P=0.0363; two-tailed unpaired t test). Each dot represents data obtained from one mouse. Size bars: 5 μm (a, left), 1 μm (a, right), and 10 μm (b). Source data are provided as a Source Data file.

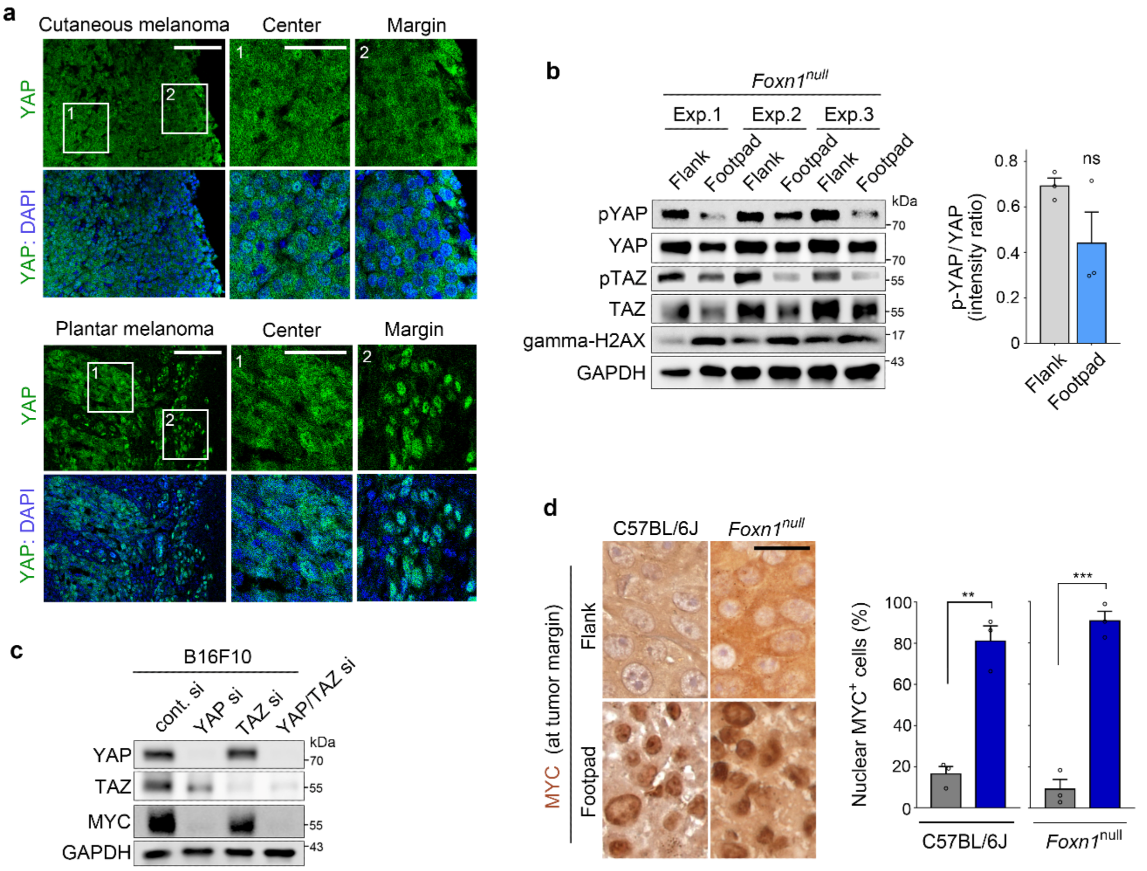

Supplementary figure 6: Influence of melanoma occurrence site and intratumoral location on the distribution YAP and S127 phosphorylation. **a**, YAP immunofluorescence staining in human nodular melanomas resected from the cheek (cutaneous) and the heel of the foot (plantar). The images shown are representative analysis results of three patient samples per group. **b**, Immunoblot analysis of the indicate proteins. The bar graph shows the intensity ratio of phosphorylated YAP (S127) and total YAP. Error bars are SEM (n=3 mice; two-tailed unpaired t test). **c**, Immunoblot analysis of MYC after knockdown of YAP and TAZ. Data shown are representative results of two independent experiments. **d**, Detection and quantification of MYC expression at the margin of flank and footpad tumors generated in C57BL/6J or *Foxn1*-null mice. Error bars are SEM (n=3 tumor pairs for each genotype; **P=0.0014 and ***P=0.0003; two-tailed unpaired t test). Each dot represents data obtained from one mouse. Size bars: 100 µm (a, left), 50 µm (a, right), and 20 µm (d). Source data are provided as a Source Data file.

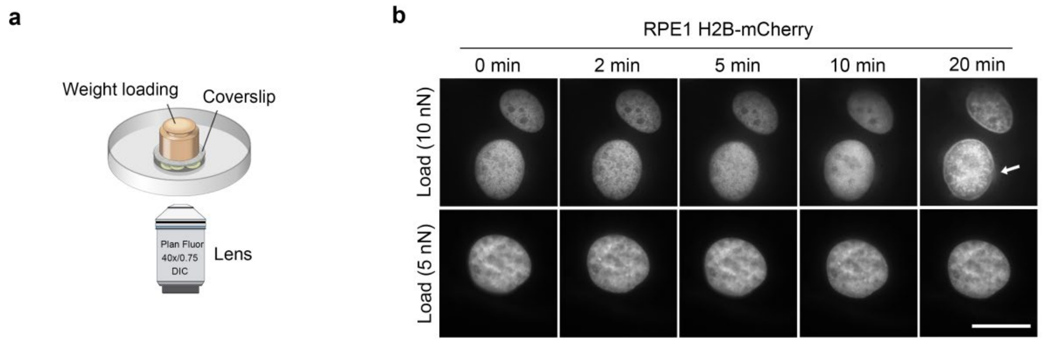

Supplementary figure 7: Experimental model for applying a compressive load to cells in culture. a, Schematic representation of the setup used to perform in vitro compressive load experiments. **b**, Live imaging of RPE1 cells stably expressing H2B-mCherry. The mechanical stress endured by the nuclear envelope of RPE1 cells, which is a normal human cell line immortalized by telomerase overexpression, was measured as a reference data. Weight loading was applied as indicated. Arrow indicates nuclear envelope damage. Representative images of two independent experiments are shown. Size bar: 10 μm (b).

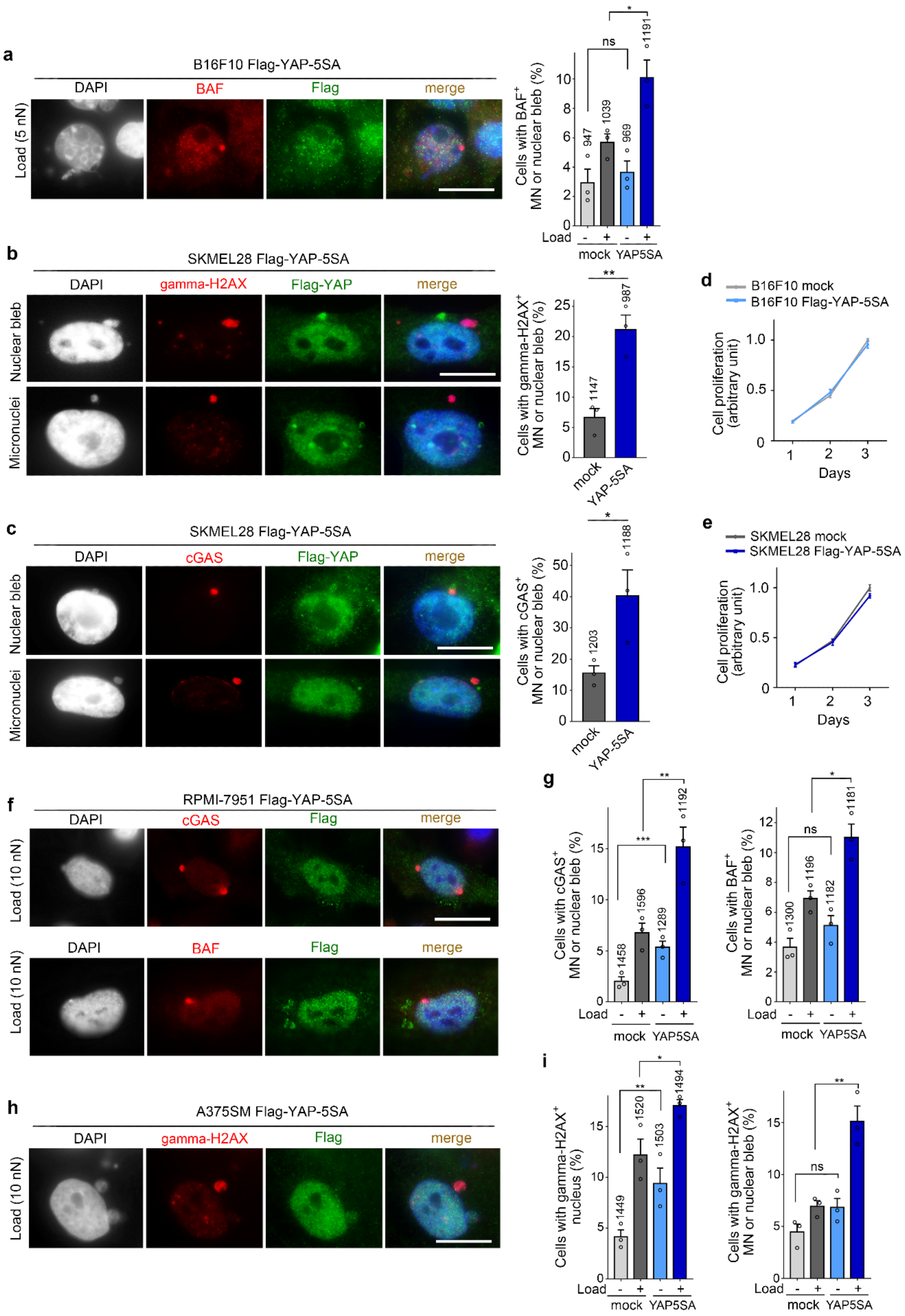

a
B16F10 Flag-YAP-5SA
DAPI
BAF
Flag
merge
Load (5 nN)
Cells with BAF+ MN or nuclear bleb (%)
Load
mock
YAP5SA
ns
947
1039
969
1191
b
SKMEL28 Flag-YAP-5SA
DAPI
gamma-H2AX
Flag-YAP
merge
Nuclear bleb
Micronuclei
Cells with gamma-H2AX+ MN or nuclear bleb (%)
mock
YAP-5SA
1147
987
c
SKMEL28 Flag-YAP-5SA
DAPI
cGAS
Flag-YAP
merge
Nuclear bleb
Micronuclei
Cells with cGAS+ MN or nuclear bleb (%)
mock
YAP-5SA
1203
1188
d
B16F10 mock
B16F10 Flag-YAP-5SA
Cell proliferation (arbitrary unit)
Days
e
SKMEL28 mock
SKMEL28 Flag-YAP-5SA
Cell proliferation (arbitrary unit)
Days
f
RPMI-7951 Flag-YAP-5SA
DAPI
cGAS
Flag
merge
Load (10 nN)
DAPI
BAF
Flag
merge
Load (10 nN)
g
Cells with cGAS+ MN or nuclear bleb (%)
1458
1596
1289
1192
Load
mock
YAP5SA
Cells with BAF+ MN or nuclear bleb (%)
ns
1300
1196
1182
1181
Load
mock
YAP5SA
h
A375SM Flag-YAP-5SA
DAPI
gamma-H2AX
Flag
merge
Load (10 nN)
i
Cells with gamma-H2AX+ nucleus (%)
1449
1520
1503
1494
Load
mock
YAP5SA
Cells with gamma-H2AX+ MN or nuclear bleb (%)
ns
Load
mock
YAP5SA

Supplementary figure 8: Nuclear membrane instability of melanoma cell lines expressing YAP-5SA. **a,** BAF immunofluorescence staining in B16F10 Flag-YAP-5SA cells, and quantification BAF^{+} micronucleus (MN)/nuclear blebs. Cells were subjected to weight loading (5 nN) for 15 min (*P=0.0289). **b**, Gamma-H2AX immunofluorescence staining in SKMEL28 Flag-YAP-5SA cells, and quantification of gamma-H2AX^{+} MN/nuclear blebs (**P=0.007). **c**, cGAS immunofluorescence staining in SKMEL28 Flag-YAP-5SA cells, and quantification cGAS^{+} MN/nuclear blebs (*P=0.045). **d**, Cell proliferation analysis (in triplicate) of the indicated B16F10 cells. **e**, Cell proliferation analysis (in triplicate) of the indicated SKMEL28 cells. **f**, cGAS and BAF immunofluorescence staining in RPMI-7951 cells stably expressing Flag-YAP-5SA. Cells were subjected to weight loading (10 nN) for 15 min. **g,** Quantification of cGAS^{+} and BAF^{+} MN/nuclear blebs in RPMI-7951 Flag-YAP-5SA cells (left, ***P=0.01 and **P=0.0169; right, *P=0.0171). **h**, Gamma-H2AX immunofluorescence staining in A375SM cells stably expressing Flag-YAP-5SA. Cells were subjected to weight loading (10 nN) for 15 min. **i**, Quantification of gamma-H2AX^{+} nuclei and gamma-H2AX^{+} MN/nuclear blebs in A375SM cells (left, **P=0.0341 and *P=0.044; right, **P=0.0066). Error bars are SEM (n=3 independent experiments; two-tailed unpaired t test). Each dot represents data from one independent experiment. The number above each bar is the total number of cells analyzed. The two graphs in i are quantifications for the same cells. Size bars: 10 μm (a, b, c, f and h). Source data are provided as a Source Data file.

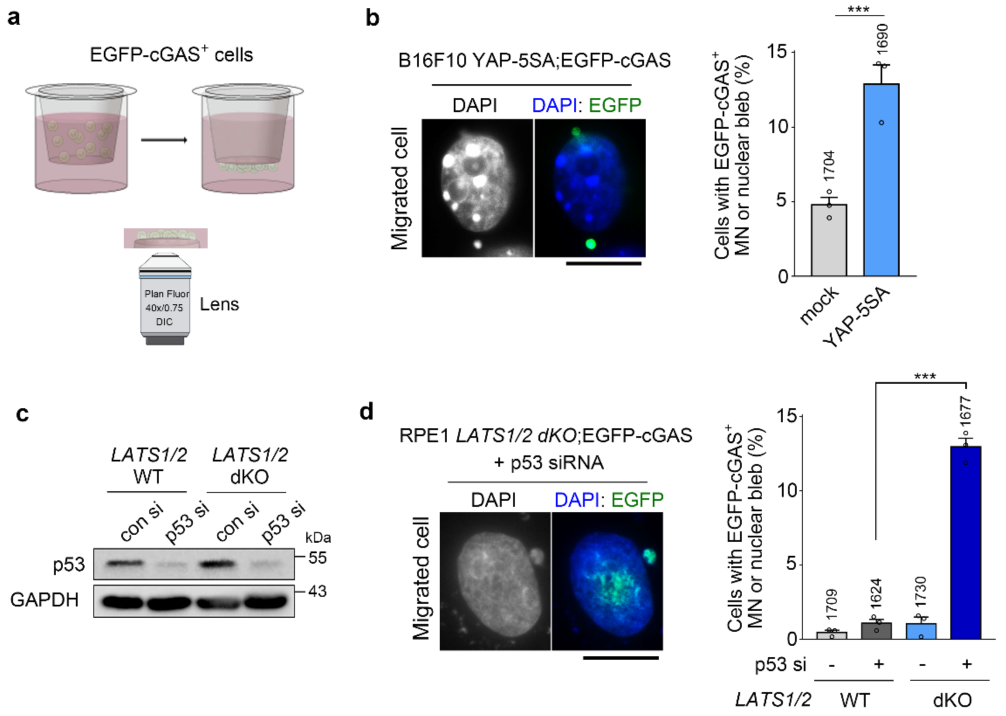

Supplementary figure 9: Nuclear membrane rupture during transwell migration of YAP-activated cells. **a**, Schematic diagram of the transwell migration assay. **b**, Detection and quantification of EGFP+ MN/nuclear blebs in B16F10 cells expressing YAP-5SA and EGFP-cGAS. EGFP and DAPI fluorescence was imaged in cells that passed through the transwell (***P=0.0043). **c**, Immunoblot analysis confirming knockdown of p53 genes. **d**, Detection and quantification of EGFP+ MN/nuclear blebs in RPE1 LATS1/2 wildtype or null cells expressing EGFP-cGAS after knockdown of the p53 gene (***P<0.0001). Error bars are SEM (n=3 independent experiments; two-tailed unpaired t test). Each dot represents data from one independent experiment. The number above each bar is the total number of cells analyzed. Size bars: 10 μm (b and d). Source data are provided as a Source Data file.

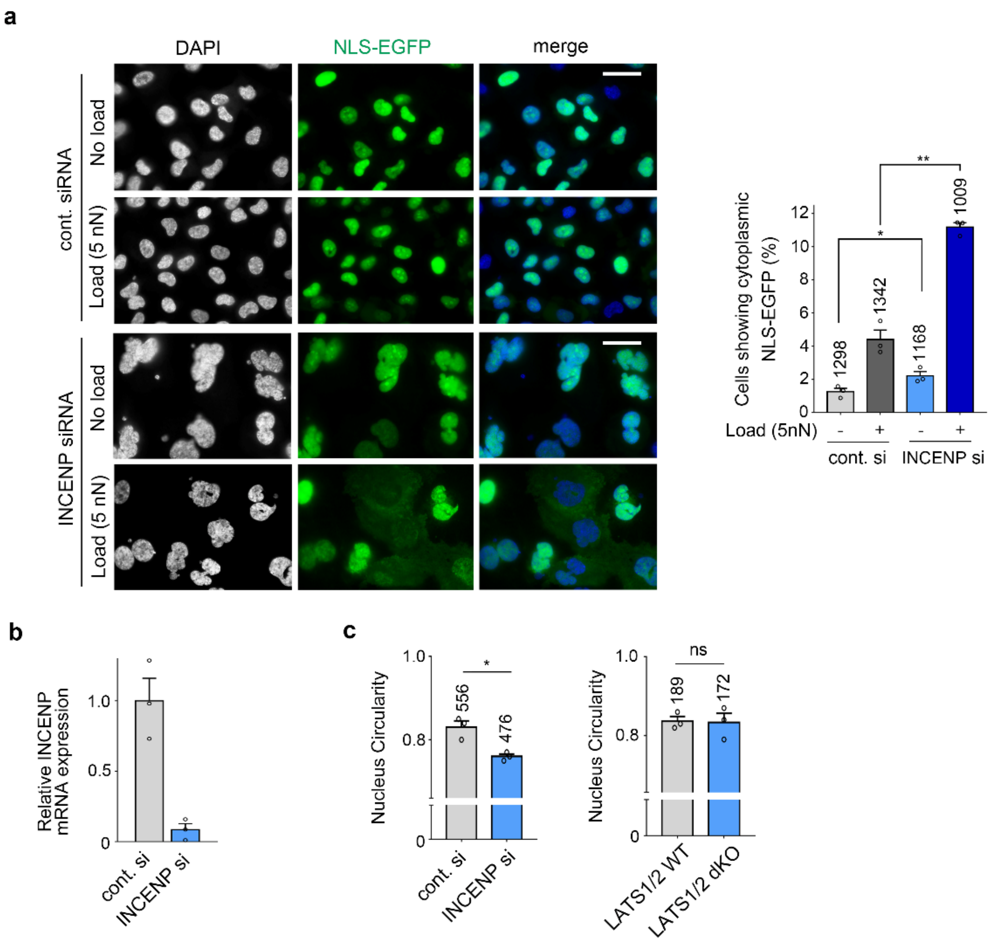

Supplementary figure 10: Correlation between morphological abnormalities of the nucleus and its susceptibility to weight loading. a, Detection and quantification of nuclear envelope rupture by monitoring the release of EGFP tagged with a nuclear localization sequence (NLS-EGFP) to the cytoplasm (*P=0.0442 and **P=0.0004). RPE1 NLS-EGFP cells were transfected with INCENP siRNAs 48 hrs before weight loading for 20 min. **b**, Quantitative RT-PCR analysis (in triplicate) for measuring the *INCENP* mRNA level 48 hrs after siRNA transfection. **c**, Quantification of nuclear circularity in INCENP-depleted RPE1 cells and RPE1 LATS1/2dKO cells (*P=0.0128). Error bars are SEM (n=3 independent experiments; two-tailed unpaired t test). Each dot represents data from one independent experiment. The number above each bar is the total number of cells analyzed. Size bars: 20 μm. Source data are provided as a Source Data file.

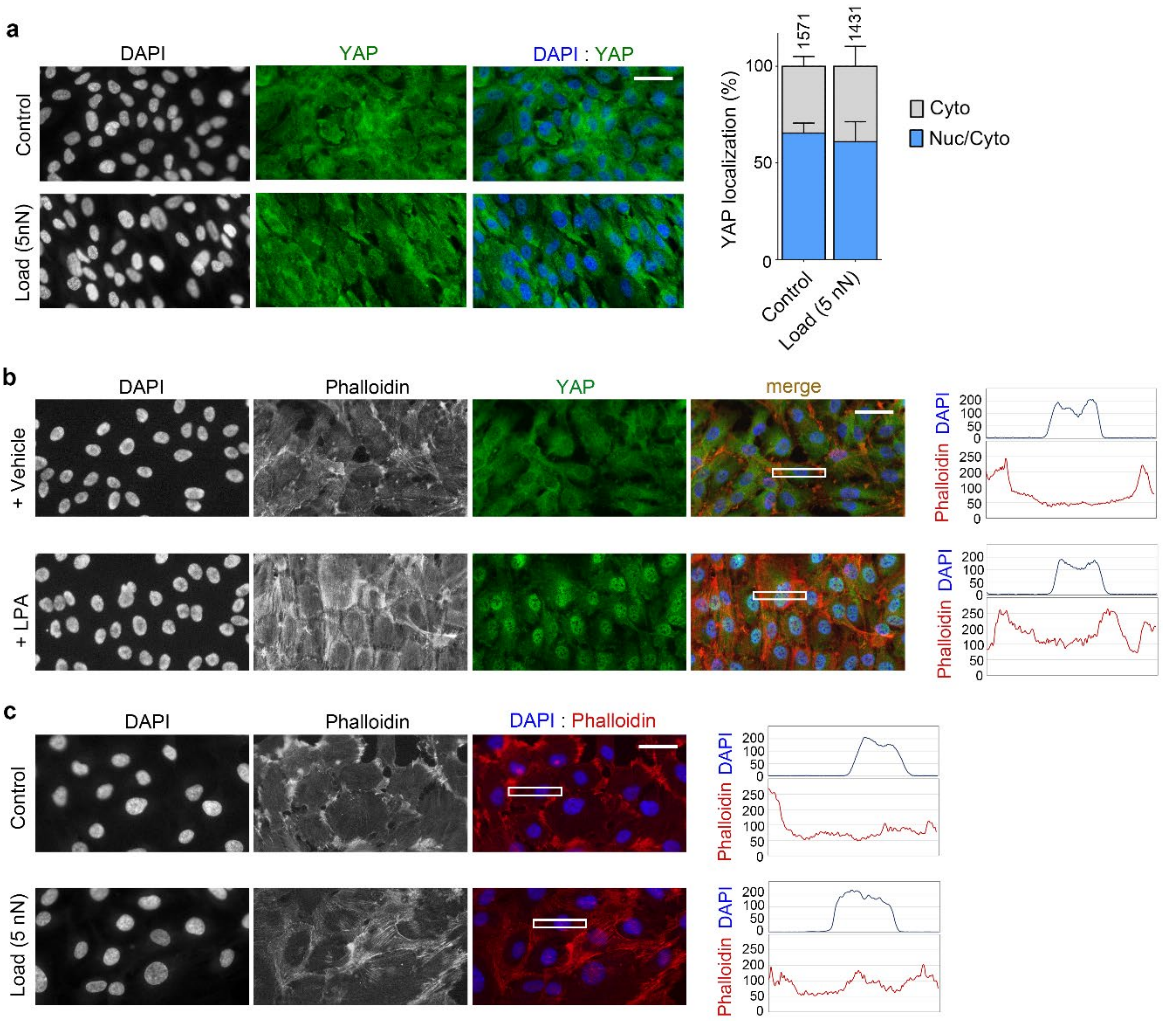

Supplementary figure 11: Effect of compressive loading on YAP localization and actin filament assembly. a. Immunofluorescence analysis of YAP localization in confluent RPE1 cell layers subjected to weight loading (5 nN) for 20 min. The bar graph is quantification of the YAP localization pattern [Nuc/Cyto, equivalent YAP distribution in the nucleus and cytoplasm; and Cyto, cytoplasmic localization of YAP]. Error bars are SEM (n=3 independent experiments). The number above each bar is the total number of cells analyzed. **b.** Staining of RPE1 cells with phalloidin-dye conjugates and YAP antibodies. Cells were cultured in serum free media for 12 hrs before LPA treatment at 20 μM for 30 min. The graphs illustrate the quantification in arbitrary units of the distribution of DAPI and phalloidin fluorescence in the boxed area. **c.** Phalloidin labeling of RPE1 cells subjected to weight loading (5 nN) for 20 min. Size bars: 20 μm (a-c). Source data are provided as a Source Data file.

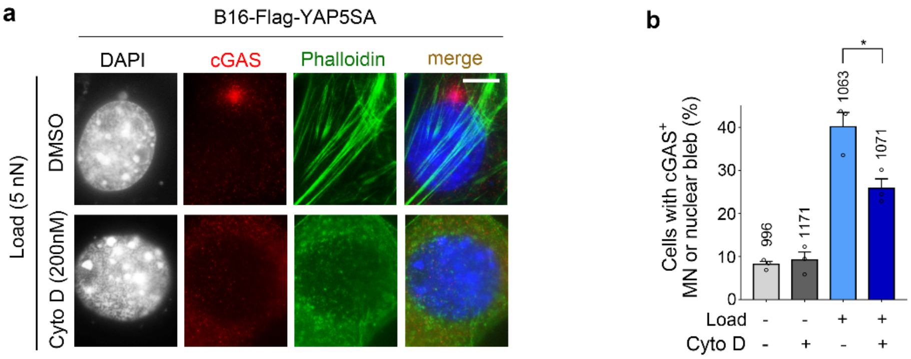

Supplementary figure 12: Effect of actin filament destabilization on weight load-mediated nuclear envelope rupture in melanoma cells expressing YAP-5SA. a, B16-Flag-YAP5SA cells were subjected to weight loading for 20 min in the presence of 200 nM cytochalasin D. Cells were stained with anti-cGAS antibody and fluorescently labeled phalloidin. **b,** Quantification of micronucleus (MN)/nuclear bleb rupture in B16-Flag-YAP5SA cells treated as indicated. Error bars are SEM (n=3 independent experiments; *P=0.0227; two-tailed unpaired t test). The number above each bar is the total number of cells analyzed. Size bar: 5 μm (a). Source data are provided as a Source Data file.

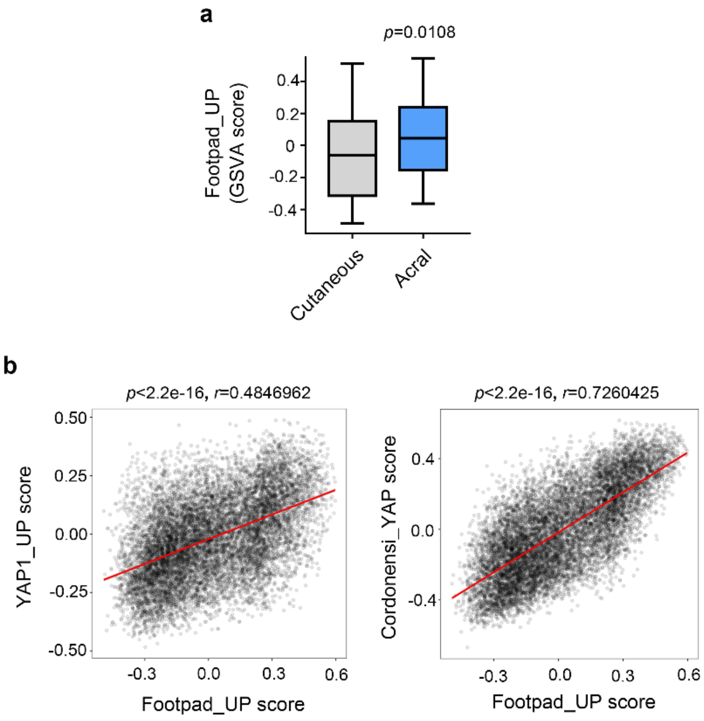

Supplementary figure 13: Gene set variation analysis (GSVA) for the footpad_up signature using public data sets of human cancer samples. a, GSVA scores showing that the expression of the upregulated genes in mouse footpad tumors (footpad_up gene set) is higher in acral melanoma than in cutaneous melanoma (n=145 cutaneous melanoma samples; n=43 acral melanoma samples). The box plot indicates median (middle line), 25th and 75th percentile (box), and 1st and 99th percentile (whiskers). Statistical significance was tested by the Tukey method. **b**, Positive correlation between GSVA scores of footpad_up signature and YAP signatures (YAP1_UP and Cordonensi_YAP_Conserved; GSEA) in human cancer. The analysis was performed using 11,023 TCGA cases across 33 cancer types. TCGA data were analyzed by a Pearson's product-moment correlation test (two-tailed P value<2.2e-16; r=Pearson's correlation coefficient). Source data are provided as a Source Data file.

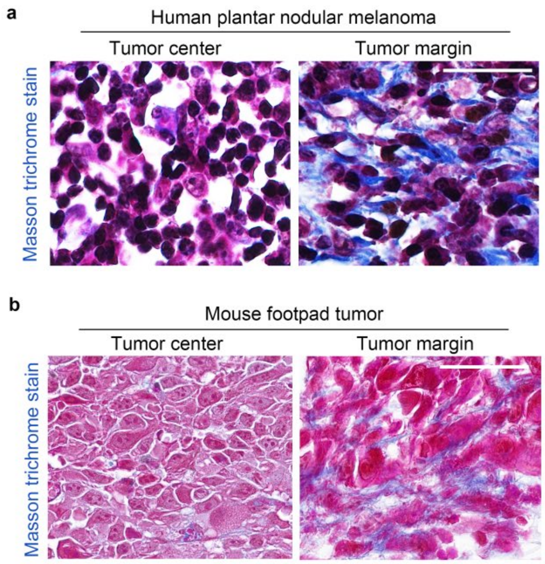

Supplementary figure 14. Influence of the location within tumor nodules on the level of extracellular matrix collagen expression. Masson trichrome staining shows elevated levels of collagen fibers in the marginal region of a human nodular melanoma resected from the heel of the foot (**a**) and a mouse footpad tumor (**b**). The images shown are representative analysis results of three melanoma patients and three mice. Size bars: 50 μm (a and b).

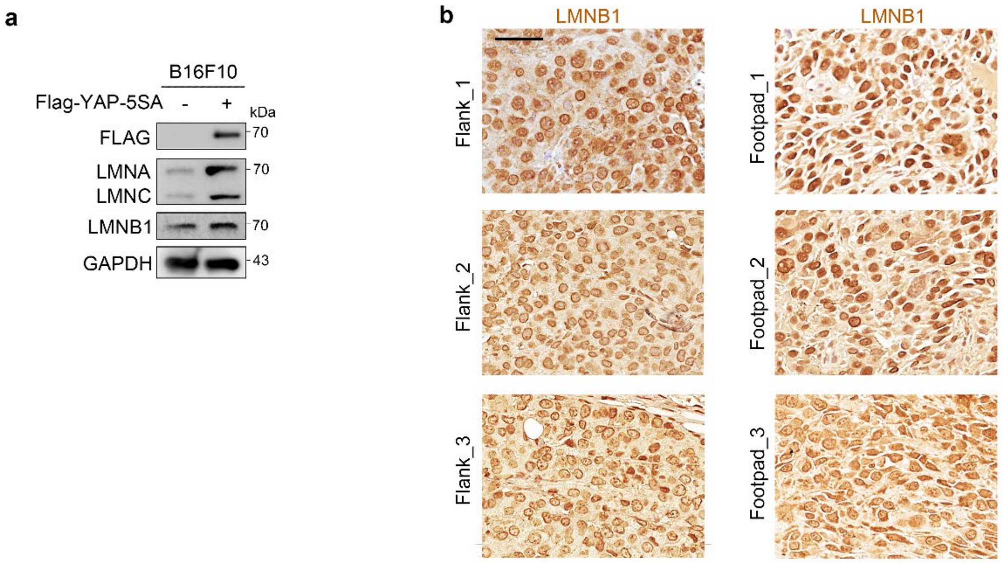

Supplementary figure 15: Influence of YAP activity and melanoma occurrence site on lamin protein expression. a, Immunoblot analysis of lamin A/C (LMNA/LMNC) and lamin B1 (LMNB1) expression in B16F10 cells stably expressing Flag-YAP-5SA. Data shown are representative results of three independent experiments. **b**, Immunohistochemistry analysis of LMNB1 expression in B16F10 cells stably expressing Flag-YAP-5SA implanted into the flank and the footpad of C56BL/6J mice (n=3 mice). Size bar: 50 μm (b). Source data are provided as a Source Data file.

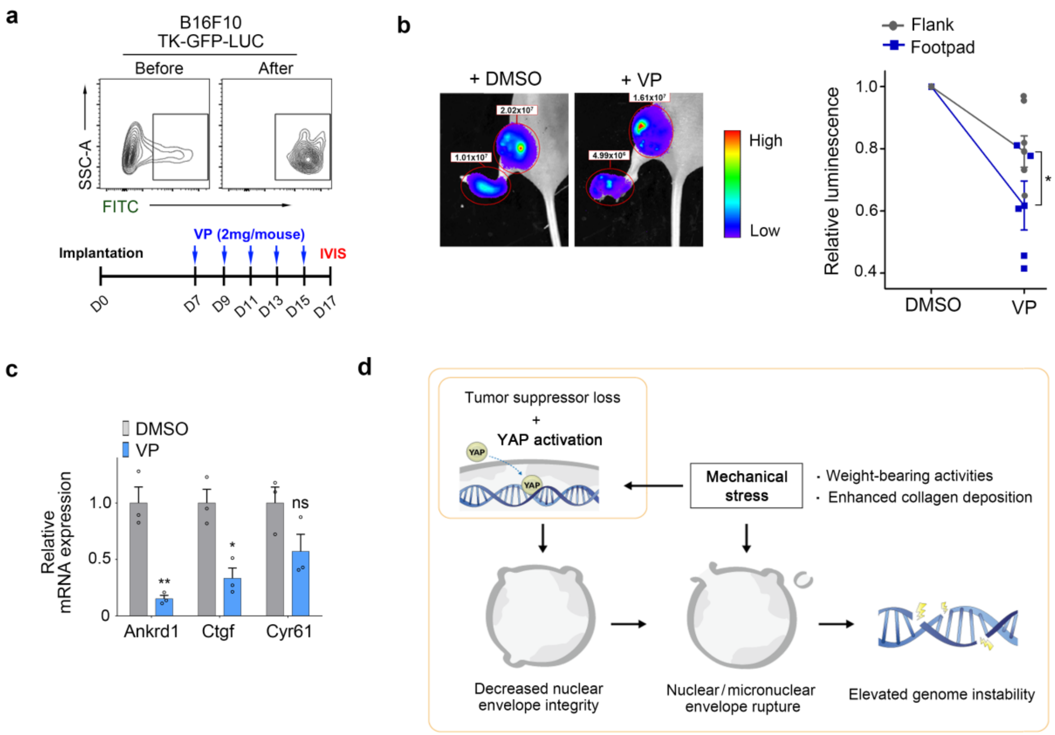

Supplementary figure 16: Inhibition of tumor growth by verteporfin. a, Implantation of B16F10 cells expressing EGFP and luciferase, and time points of verteporfin injection. B16F10 cells transfected with TK-GFP-LUC vector were sorted based on EGFP fluorescence before injection into C57BL/6J mice. **b**, In vivo bioluminescence imaging of implanted tumor cells, and quantification of luminescence. Error bars are SEM (n=6 mice; *P=0.0154; two-tailed unpaired t test). **c**, Quantitative RT-PCR analysis for measuring mRNA levels of YAP target genes after verteporfin treatment as in a. Error bars are SEM (n=3 mice; **P=0.0042 for Ankrd1 and *P=0.0117 for Ctgf; two-tailed unpaired t test). **d**, Summary of the processes by which mechanical stress induces instability of the nuclear membrane and genome. Source data are provided as a Source Data file.
