# Supplementary material for: Weight-bearing activity impairs nuclear membrane and genome integrity via YAP activation in plantar melanoma
Source: Nat Commun. 2022 Apr 25;13:2214. doi: 10.1038/s41467-022-29925-x (PMC9038926; doi:10.1038/s41467-022-29925-x)
Supplement: Supplementary file 2 — Reporting summary [file 41467_2022_29925_MOESM2_ESM.pdf]

nature portfolio

Corresponding author(s): Joon Kim, Kee Yang Chung, Mi Ryung Roh

Last updated by author(s): Mar 10, 2022

Reporting Summary

Nature Portfolio wishes to improve the reproducibility of the work that we publish. This form provides structure for consistency and transparency in reporting. For further information on Nature Portfolio policies, see our Editorial Policies and the Editorial Policy Checklist.

Statistics

For all statistical analyses, confirm that the following items are present in the figure legend, table legend, main text, or Methods section.

n/a	Confirmed	
☐	☒	The exact sample size (*n*) for each experimental group/condition, given as a discrete number and unit of measurement
☐	☒	A statement on whether measurements were taken from distinct samples or whether the same sample was measured repeatedly
☐	☒	The statistical test(s) used AND whether they are one- or two-sided<br>*Only common tests should be described solely by name; describe more complex techniques in the Methods section.*
☐	☒	A description of all covariates tested
☒	☐	A description of any assumptions or corrections, such as tests of normality and adjustment for multiple comparisons
☐	☒	A full description of the statistical parameters including central tendency (e.g. means) or other basic estimates (e.g. regression coefficient) AND variation (e.g. standard deviation) or associated estimates of uncertainty (e.g. confidence intervals)
☐	☒	For null hypothesis testing, the test statistic (e.g. *F*, *t*, *r*) with confidence intervals, effect sizes, degrees of freedom and *P* value noted<br>*Give P values as exact values whenever suitable.*
☒	☐	For Bayesian analysis, information on the choice of priors and Markov chain Monte Carlo settings
☒	☐	For hierarchical and complex designs, identification of the appropriate level for tests and full reporting of outcomes
☐	☒	Estimates of effect sizes (e.g. Cohen's *d*, Pearson's *r*), indicating how they were calculated

Our web collection on statistics for biologists contains articles on many of the points above.

Software and code

Policy information about availability of computer code

Data collection

The following software were used for data collection:
AMT Image Capture Engine software 7.0.1.329 (AMT Imaging)
Living Image 2.60 software (PerkinElmer)
Pannoramic Scannner software 2.1.2.104194 (3DHISTECH)
Softworx Deltavision software 4.1.2 (Applied Precision)
Zen 2.3 software (Carl Zeiss)

Data analysis

The following software were used for data analysis:
BWA-MEM 0.7.17-r1188 (http://bio-bwa.sourceforge.net/)
CaseViewer 2.2 (3DHISTECH)
Cellprofiler 4.2.1 (Broad Institute)
Delly 0.76 (https://github.com/dellytools/delly)
KEGG pathway database 20200706 (https://www.kegg.jp/kegg/pathway.html)
Gene Set Enrichment Analysis 4.1.0 (Broad Institute)
GraphPad Prism 7.0 (GraphPad Software)
HISAT2 2.1.0 (http://daehwankimlab.github.io/hisat2/)
ImageJ software 1.53a (NIH)
Integrative Genomics Viewer 2.8.7 (Broad institute)
Living Image 2.60 software (PerkinElmer)
Mutect2 4.1.4.1 (https://gatk.broadinstitute.org/hc/en-us/articles/360037593851-Mutect2)
Strelka 2.9.9 (https://github.com/Illumina/strelka)
R: The R Project for Statistical Computing 4.04 (https://www.r-project.org/)
R package 'circlize' 0.4.14
R package ‘GSVA’ 1.38.2

R package 'Deseq2' 1.34.0
Zen 2.3 software (Carl Zeiss)

For manuscripts utilizing custom algorithms or software that are central to the research but not yet described in published literature, software must be made available to editors and reviewers. We strongly encourage code deposition in a community repository (e.g. GitHub). See the Nature Portfolio guidelines for submitting code & software for further information.

Data

Policy information about availability of data

All manuscripts must include a data availability statement. This statement should provide the following information, where applicable:

- Accession codes, unique identifiers, or web links for publicly available datasets
- A description of any restrictions on data availability
- For clinical datasets or third party data, please ensure that the statement adheres to our policy

RNA-seq data generated in this study has been deposited in the Gene Expression Omnibus (GEO) database repository and are available under accession number GSE192835 [https://www.ncbi.nlm.nih.gov/geo/query/acc.cgi?acc=GSE192835]. Deep DNA sequencing data generated in this study has been deposited in the NCBI Sequence Read Archive (SRA) database under accession number PRJNA799072 [https://www.ncbi.nlm.nih.gov/bioproject/?term=PRJNA799072]. All other data are available in the main text or in the supplementary materials. Public datasets were obtained from publicly accessible repositories: bioRxiv [doi.org/10.1101/2020.11.14.383083], dbGap phs001486 [https://www.ncbi.nlm.nih.gov/projects/gap/cgi-bin/study.cgi?study_id=phs001486.v3.p3], TCGA Research Network [https://gdc.cancer.gov/about-data/publications/pancanatlas]. The genomic coordinates of human and mice were obtained from publicly accessible repositories: GRCh37 [https://www.ncbi.nlm.nih.gov/assembly/GCF_000001405.13/]; mm10 [https://www.ncbi.nlm.nih.gov/assembly/GCF_000001635.20/]. Source data are provided with this paper.

Field-specific reporting

Please select the one below that is the best fit for your research. If you are not sure, read the appropriate sections before making your selection.

☒ Life sciences ☐ Behavioural & social sciences ☐ Ecological, evolutionary & environmental sciences

For a reference copy of the document with all sections, see nature.com/documents/nr-reporting-summary-flat.pdf

Life sciences study design

All studies must disclose on these points even when the disclosure is negative.

Sample size	All human tissue blocks (n=26) approved by the IRB of Severance Hospital were used for analysis. Mouse cancer transplantation experiments were performed using the minimum number of individuals (3-5) in each treatment group that passed the review by the KAIST Animal Ethics Committee. For mouse experiments, sample size was chosen in accordance with previously published experiments conducted (Seo J., Cancer Research, 2019).
Data exclusions	No data were excluded.
Replication	All the experiments were repeated at least three times. All attemtps at experimental replication were successful.
Randomization	Animals from different cages, but within the same experimental group, were selected to assure randomization. For the in vitro experiments, the cell lines from one passage were seeded in different wells for further interventions.
Blinding	Analysis of human sample was performed blindly. In the case of mouse tissue, the appearance of tumor cells implanted in the skin of the footpad and flank was signficanlty different, so the blinded evaluation had no practical meaning. For other experiments, the investigators were blinded to allocation during experiments and outcome analyse.

Reporting for specific materials, systems and methods

We require information from authors about some types of materials, experimental systems and methods used in many studies. Here, indicate whether each material, system or method listed is relevant to your study. If you are not sure if a list item applies to your research, read the appropriate section before selecting a response.

Materials & experimental systems

n/a	Involved in the study
☐	☒ Antibodies
☐	☒ Eukaryotic cell lines
☐	☒ Palaeontology and archaeology
☐	☒ Animals and other organisms
☐	☒ Human research participants
☒	☐ Clinical data
☒	☐ Dual use research of concern

Methods

n/a	Involved in the study
☒	☐ ChIP-seq
☒	☐ Flow cytometry
☒	☐ MRI-based neuroimaging

Antibodies

Antibodies used

"Supplementary Table 2" of Supplementary information: The following primary and secondary antibodies were used in the immunoblotting: anti-c-MYC(Rabbit, Abcam, ab32072), anti-FLAG(Mouse, Sigma-Aldrich, F1804), anti-gamma-H2AX(Rabbit, Cell Signaling Technology, anti-GAPDH(Mouse, Santa Cruz Biotechnology, sc59540), anti-Lamin A/C (Rabbit, Cell Signaling Technology, 2032), anti-Lamin B1(Rabbit, Abcam, ab16048), anti-LATS1(Rabbit, Cell Signaling Technology, 3477), anti-LATS2(Rabbit, Cell Signaling Technology, 5888), anti-P53(Mouse, Santa Cruz Biotechnology, sc126), anti-phospho-TAZ(Rabbit, Cell Signaling Technology, 59971), anti-phospho-YAP(Ser127)(Cell Signaling Technology, 4911), anti-TAZ(Rabbit, Cell Signaling Technology, 83669), anti-YAP(Mouse, Santa Cruz Biotechnology, sc101199); and anti-mouse IgG-, anti-rabbit IgG-HRP-linked antibodies(Cell Signaling Technology, 7076; Cell Signaling Technology, 7074) were purchased from Cell Signaling Technology.

The following primary and secondary antibodies were used in the immunofluorescence: Alexa Fluor™ 488 Phalloidin (Invitrogen, A12379), Alexa Fluor™ 594 Phalloidin(Invitrogen, A12381), anti-BAF(Rabbit, Abcam, ab129184), anti-cGAS(Rabbit, Cell Signaling Technology, 15102), Mouse specific anti-cGAS(Rabbit, Cell Signaling Technology, 31659), anti-FLAG(Mouse, Sigma-Aldrich, F1804), anti-FLAG(Rabbit, Sigma-Aldrich, F7425), anti-Lamin A/C(Mouse, Santa Cruz Biotechnology, sc7292), anti-LaminB1(Rabbit, Abcam, ab16048), anti-gamma-H2AX(Rabbit, Cell Signaling Technology, 2577), anti-YAP(Mouse, Santa Cruz Biotechnology, sc101199), anti-YAP(Rabbit, Cell Signaling Technology, 14074); and Alexa Flour 488-, Alexa Flour 594-conjugated anti-mouse, anti-rabbit secondary antibodies(Invitrogen, A-21202; Invitrogen, A-21203; Invitrogen, A-21207) were purchased from Invitrogen. Nuclei were stained with DAPI (Sigma).

The following primary and secondary antibodies were used in the immunohistochemistry: anti-gamma-H2AX(Rabbit, Cell Signaling Technology, 2577), anti-Lamin B1(Rabbit, Abcam, ab16048), anti-Myc(Rabbit, Millipore, 06-340), anti-YAP(Mouse, Santa Cruz Biotechnology, sc101199), anti-YAP(Mouse, Santa Cruz Biotechnology, sc101199), anti-YAP(Rabbit, Cell Signaling Technology, 14074); and biotinylated anti-mouse, anti-rabbit IgG conjugated secondary antibodies (Vector laboratories, BA-2000-1.5; Vectorl aboratories, BA-1000-1.5) were purchased from Vector Laboratories. Sections were counterstained with Harris hematoxylin (Papanicolaou solution 1a, Merck).

Validation

All the antibodies were validated for the species (human or mouse) and applications (immunoblotting, immunofluorescence and immunohistochemistry) by the correspondent manufacturer, which is described in the manufacturer's website. Our usage was described in the Methods section of the manuscript as below.

Immunoblotting: Membranes were incubated with different primary antibodies in TBST buffer with 5% BSA or skim milk. After incubation, membranes were washed with TBST buffer, incubated with anti-mouse IgG-, anti rabbit IgG-HRP-linked secondary antibodies (1:2000 dilution) at 4 °C for 1 hours. Target proteins were detected using enhanced chemiluminescence western blot detection solution (LumiGlo, KPL; Western Bright, Advansta).

Immunofluorescence: Cells were fixed with 4 % paraformaldehyde (PFA) for 8 min at room temperature (RT). After fixation, 0.1 % Triton X-100 (Sigma-Aldrich) was applied for permeabilization. Cells were incubated with primary antibodies for 1 hr at RT. Cells were incubated with Alexa Fluor 488- or 594-conjugated secondary antibodies (Life Technologies) for 1 hr at RT. For staining actin filaments, Alexa Fluor 488- or 594-conjugated Phalloidin (Life Technologies) was used according to manufacturer's protocol. For immunofluorescence staining of the mouse tumor, tumor tissues were fixed in 4 % PFA overnight in 4 °C, dehydrated in 30 % sucrose solution, and embedded in tissue freezing medium (Leica). Cryosections were blocked with 3 % donkey serum in PBST (0.3 % Triton X-100 in PBS) and then incubated at 4 °C overnight with primary antibodies. The samples were washed five times with PBS, followed by incubation with secondary antibodies for 2 hr at 4 °C (Invitrogen, 1:1000 dilution).

Immunofluorescence of paraffin sections: After deparaffinization and heat-induced antigen retrieval, samples were blocked with 3 % donkey serum in PBST (0.3% Triton X-100 in PBS) and then incubated at 4°C overnight with primary antibodies. After several washes, the samples were incubated for 2 hr at 4°C with Alexa Fluor 488- or 594-conjugated secondary antibodies (Invitrogen; 1:1000 dilution). To eliminate the autofluorescence, autofluorescence quenching agent (TrueVIEW, Vector Laboratories) was used according to manufacturer's instructions. Sections were mounted with anti-fade mounting medium (Vibrance, Vector Laboratories).

Immunohistochemistry: Paraffin sections were deparaffinized and antigen-retrieved using a pressure cooker. Sodium-citrate buffer (pH 6) or Tris-EDTA buffer (pH 9) were used as an antigen retrieval solution, depending on the type of the antigen. The Sections were incubated with BLOXALL (Vector Laboratories) at RT, blocked with 3 % horse (or goat) serum in PBS or PBST (0.3% Triton X-100 in PBS), and then incubated at 4 °C overnight with primary antibodies. The samples were washed and incubated with biotinylated IgG secondary antibody (Vector Laboratories; 1:200 dilution) for 30 min at RT. VECTASTAIN ABC kit and DAB was used according to the manufacturer's protocol (Vector Laboratories). Sections were counterstained with Harris hematoxylin (Papanicolaou solution 1a, Merck) and mounted with mounting medium (DAKO).

Eukaryotic cell lines

Policy information about cell lines

Cell line source(s)	"Cell culture and reagents" of the "Methods": A375SM(Korean Cell Line Bank; 80004), B16F10(ATCC; CRL-6475™), HEK293T(ATCC; CRL-3216™), SKMEL28(ATCC; HTB-72™), RPE1(ATCC; CRL-4000™), RPMI-7951(ATCC; HTB-66™).
Authentication	Authentications of cells were based on their morphology, growth condition and specific gene expression.
Mycoplasma contamination	All cells were confirmed to be mycoplasma-negative by DAPI staining.
Commonly misidentified lines (See ICLAC register)	No commonly misidentified cell lines were used in the study.

Palaeontology and Archaeology

Specimen provenance	*Provide provenance information for specimens and describe permits that were obtained for the work (including the name of the issuing authority, the date of issue, and any identifying information). Permits should encompass collection and, where applicable, export.*
Specimen deposition	*Indicate where the specimens have been deposited to permit free access by other researchers.*
Dating methods	*If new dates are provided, describe how they were obtained (e.g. collection, storage, sample pretreatment and measurement), where they were obtained (i.e. lab name), the calibration program and the protocol for quality assurance OR state that no new dates are provided.*

☐ Tick this box to confirm that the raw and calibrated dates are available in the paper or in Supplementary Information.

Ethics oversight	*Identify the organization(s) that approved or provided guidance on the study protocol, OR state that no ethical approval or guidance was required and explain why not.*

Note that full information on the approval of the study protocol must also be provided in the manuscript.

Animals and other organisms

Policy information about studies involving animals; ARRIVE guidelines recommended for reporting animal research

Laboratory animals	"Cell implantation in mice" of "Methods": C57BL/6J mice (6 weeks, female), Foxn1-null mice(6 weeks, female) were used for experiments. All mice were housed in an approved animal facility at KAIST under a 12 h light-dark cycle and 50 ± 10 % humidity and a temperature of 22 ± 2 °C. Mice were allowed ad libitum access to a standard diet (PMI LabDiet) and water.
Wild animals	The study did not involve wild animals.
Field-collected samples	The study did not involve field-collected samples.
Ethics oversight	"Study Approval" of "Methods": The KAIST IACUC approved the animal care and experimental procedures used in this study (KA2017-26). The maximum allowable tumor size (20 mm in diameter) was not exceeded.

Note that full information on the approval of the study protocol must also be provided in the manuscript.

Human research participants

Policy information about studies involving human research participants

Population characteristics	Human melanoma specimens were collected from patients who underwent surgery at Severance Hospital. Characteristics of patients are described in "Supplementary table 1" of supplmentary information.
Recruitment	We collected the human specimens in patients who received surgery. Our study fits the category of expedited human study as there is no additional risk to participants. There was no compensation for participants.
Ethics oversight	"Study Approval" of "Methods": The human sample experiments were reviewed and approved by the institutional review board at Severance Hospital (IRB: 2020-2401-002). Only essential information about human participants collected with informed consent is disclosed.

Note that full information on the approval of the study protocol must also be provided in the manuscript.
